# Identification of potential inhibitor against *Leishmania donovani* mitochondrial DNA primase through in-silico and in vitro drug repurposing approaches

**DOI:** 10.1038/s41598-024-53316-5

**Published:** 2024-02-08

**Authors:** Mitul Nath, Deep Bhowmik, Satabdi Saha, Rajat Nandi, Diwakar Kumar

**Affiliations:** Department of Microbiology, Assam University, Silchar, Assam 788011 India

**Keywords:** Computational biology and bioinformatics, Drug discovery, Diseases

## Abstract

*Leishmania donovani* is the causal organism of leishmaniasis with critical health implications affecting about 12 million people around the globe. Due to less efficacy, adverse side effects, and resistance, the available therapeutic molecules fail to control leishmaniasis. The mitochondrial primase of *Leishmania donovani* (*LdmtPRI1*) is a vital cog in the DNA replication mechanism, as the enzyme initiates the replication of the mitochondrial genome of *Leishmania donovani*. Hence, we target this protein as a probable drug target against leishmaniasis. The de-novo approach enabled computational prediction of the three-dimensional structure of *LdmtPRI1*, and its active sites were identified. Ligands from commercially available drug compounds were selected and docked against *LdmtPRI1*. The compounds were chosen for pharmacokinetic study and molecular dynamics simulation based on their binding energies and protein interactions. The *LdmtPRI1* gene was cloned, overexpressed, and purified, and a primase activity assay was performed. The selected compounds were verified experimentally by the parasite and primase inhibition assay. Capecitabine was observed to be effective against the promastigote form of *Leishmania donovani*, as well as inhibiting primase activity. This study's findings suggest capecitabine might be a potential anti-leishmanial drug candidate after adequate further studies.

## Introduction

The intracellular protozoan parasite belonging to the genus *Leishmania* causes the consequential parasitic disease leishmaniasis, which substantially impacts human health^1^. Globally, the prevalence of *Leishmania* spp. infections have exceeded 12 million people, with 350 million individuals susceptible to infection^2^. Leishmaniasis exhibits a disproportionately higher incidence among underprivileged people in economically disadvantaged countries, affected by elimination programs with insufficient funding, minimal attention from pharma companies and inadequacies in healthcare facilities^3–5^.

Leishmaniasis is attributable to approximately 20 distinct *Leishmania* species, transmitted through various species of phlebotomine sandflies^6^. The infection spreads through female sandflies (*Phlebotomus* species) in the Old World (Asia, Africa, and Europe) and via *Lutzomyia* spp. in the New World (the Americas)^7^. The parasite subsists in two unique forms: the extracellular promastigote present in the female sandfly's lumen and the intracellular amastigote that proliferates inside the monocyte-macrophage cells of the mammalian host^8,9^. Parasite transmission unfolds through the hematophagous engagement of the sand fly, affecting the transfer of flagellated promastigotes into the human host^10,11^.

While many instances of *Leishmania* infections may proceed without apparent symptoms^12^, the clinical conditions of leishmaniasis include cutaneous leishmaniasis, mucocutaneous leishmaniasis and visceral leishmaniasis^13–15^. Visceral leishmaniasis considered the most severe form, involves the parasite infiltration of crucial organs such as the liver, spleen, and bone marrow^16^. This condition is widespread across 78 countries^17^. However, over 90% of global visceral leishmaniasis cases are collectively reported in Brazil, India, Kenya, Somalia, South Sudan, Sudan, and Ethiopia^18,19^. In India, the predominant cause of leishmaniasis was instigated by *L. donovani* transmitted via the sandfly vector *Phlebotomus argentipes*.

Duplex DNA replication is an exceedingly intricate and synchronized procedure comprising the activity of multiple enzymes together^20^. In the last few years, specific emphasis has been placed on DNA replication in subcellular organelles of eukaryotic cells^21^ and DNA replication within the cell. DNA primase enzyme is crucial in initiating DNA replication. The mitochondria of trypanosomatids contain a unique extra-chromosomal DNA, kinetoplast DNA or the kDNA, consisting of thousands of minicircles (0.5–10 kb each) and a few dozen maxicircles (20–40 kb each) topologically linked to form a catenated DNA network^22^.

The complex process of kinetoplast DNA (kDNA) replication relies on a specialized set of primase enzymes, PRI1 and PRI2, intricately involved in triggering the initiation of replication for both maxicircles and minicircles within kDNA of kinetoplastids. PRI1 plays a critical role in cell growth and kDNA replication, as RNA interference (RNAi) directed at PRI1 led to the depletion of maxicircle DNA^23^, aligning with PRI1's role in priming maxicircle DNA replication, encompassing the encoding of rRNA (12S and 9S) and protein-coding genes in the oxidative phosphorylation pathway. Conversely, RNA interference (RNAi) of PRI2 resulted in the loss of intermediate replication and free minicircles, indicating PRI2 is necessary for minicircle replication^24^. The replication of kDNA takes place during the S phase of the cell cycle, analogous to that of nuclear DNA replication^25^.

DNA replication stands as a fundamental event within the cell, and DNA primase's proficiency to initiate the replication of DNA is significant for the subsistence and sustenance of all organisms. The replication of the mitochondrial genome in *Leishmania* spp. follows an explicit mechanism started by a crucial enzyme known as mitochondrial primase. This enzyme is indispensable for replication initiation and facilitating the parasite's growth^26^. The significance of mitochondrial primase (PRI1) in kinetoplast replication plus essentiality for cell growth as demonstrated from pioneering work^23^, validating that loss of PRI1 results in inhibition of maxicircle DNA replication, thereby hindering cellular metabolism. Consequently, we were inclined to designate the mitochondrial primase enzyme (PRI1) as a promising candidate for drug targeting.

In drug discovery, chemical biology is employed with computational drug design techniques to facilitate the identification and optimization of lead compounds^27^. Leveraging computer-aided drug discovery (CADD) techniques in the early stages has played a crucial role in accelerating the drug discovery and development process, reducing costs, and mitigating the risk of failure in the penultimate stages^28^. Using coherent drug design by CADD gives valuable insights into interactions between molecules and binding affinity amongst proteins and ligands. High-performance computing facilities, parallel processing, and enhanced programs and algorithms have primarily contributed to discovering new drug candidates^27^.

Currently, a vaccine for leishmaniasis is absent, and the therapeutic approach is exclusively dependent on a restricted array of chemotherapeutic agents^29^. Pentavalent antimony (Sb5+) served as the conventional primary treatment, but its effectiveness was limited due to the emergence of resistance^30^. Substitute treatment options in Amphotericin B, miltefosine and paromomycin are available, but their utility is restricted as the drugs are costly, toxic and possess other side effects^31^. The absence of an economical and effective drug remains a concern, necessitating the development of novel therapeutic compounds^32^ with substantial antileishmanial effects and low toxicity to the host.

Given the formidable challenges associated with treating and controlling leishmaniasis, the imperative arises to discern and characterize new antileishmanial targets and novel pharmacological agents. This has spurred our research focus towards the mitochondrial DNA primase of *Leishmania donovani* (*LdmtPRI1*) as a prospective drug target for combating leishmaniasis; utilizing virtual screening and in-vitro methods in our research endeavours by strategically leveraging drug repurposing, we aim to introduce novel therapeutic alternatives that capitalize on the intrinsic potential of existing pharmaceutical compounds, presenting a more efficient and economically viable strategy for the management of leishmaniasis.

## Methodology

### *LdmtPRI1* structure prediction and validation

*LdmtPRI1* amino acid sequences (accession id: LdBPK_230850.1) were retrieved from the TriTrypDB database (https://tritrypdb.org/tritrypdb/app/)^33^ and were subjected to protein BLAST against human proteome. BLASTp results exhibited that primase does not possess a sequence related to any human proteins, and no crystal structure is available in the PDB database. De novo predictive models were built with a Robetta server (https://robetta.bakerlab.org/) and validated by PROCHECK^34^. Models were built employing the state-of-the-art three-track Neural Network RoseTTAfold^35^ available on the Robetta server (https://robetta.bakerlab.org/)^36^ and were validated by PROCHECK^34^. Further, the most optimal model underwent energy minimization to attain a low-energy and stable conformation, which was carried out using YASARA^37^.

### Model evaluation

Post dynamism refinement, a conformational feature of the protein model was estimated via SAVESv6.0 (https://saves.mbi.ucla.edu/) and ProSa^38^. The structural conformation of the model was verified through PROCHECK^34^.

### Active site prediction

Identifying ligand binding situates within proteins is a prerequisite for various applications in the procedure for drug design^39^. COFACTOR predicted potential ligand binding sites of *LdmtPRI1*^40^ and was further validated by the FTSite server^41^.

### Preparation of ligand coordinate files

Before the virtual screening process, ligands must be prepared to form three-dimensional shapes, determine the appropriate bond sequence, and generate available tautomeric and ionization conditions^42^. The initial requirement for a small molecule as a ligand is a stereo-chemically determined geometry with an appropriate protonation state, as the docking programme evaluates conformations concerning the binding residues within the *LdmtPRI1* target^43^. A total of 4240 approved and clinical drug compounds (Drug Repurposing Library L9200) from Targetmol (https://www.targetmol.com/) were downloaded as *structure data files* (sdf format). Chemical information regarding the compounds was retrieved along with the SMILES files from the PubChem^44^ database, which was then converted to low-energy 3D dockable compounds as a pdbqt file with CORINA Classic^45^.

### Molecular docking and interaction analysis

Molecular docking, a sophisticated computational technique at the forefront of structural biology and drug discovery, is utilized adeptly to predict and elucidate the intricate binding interactions between small molecules and target proteins. The study utilized the PyRx software, leveraging the Autodock Vina docking platform^46^ to conduct protein–ligand docking analyses. This approach facilitated the precise docking of proteins and ligands, enabling the discernment of compounds that could impede the target protein's function. Using AutoDock Vina, the macromolecule (*LdmtPRI1*) and ligands were docked into predefined binding sites within a grid box set along the X, Y, and Z axes, with dimensions of 25.43, 29.65, and 19.41 Angstrom, respectively. The docking procedure was routed to an exhaustiveness of 8 and fixed to yield a pose with the lowest energy. The best-conformity protein–ligand complexes were analyzed via Pymol molecular visualization software^47^, and Ligplot was employed to visualize the 2D interactions^34^.

### Drug likeness and molecular properties of ligands

To evaluate the molecular characteristics and drug-like properties of the ligands, we used Lipinski's (http://www.scfbio-iitd.res.in/software/drugdesign/lipinski.jsp) and Molsoft's servers (http://www.scfbio-iitd.res.in/software/drugdesign/lipinski.jsp)^44^. Lipinski's rule states that for a drug to be orally effective, it should meet at least 4 out of the 5 known norms: molecular weight, octanol/water partition coefficient, H-bond donor, H-bond acceptor, and molar refractivity index that can differentiate between the drug and non-drug molecules^48^.

### Admet profiling

Pharmacodynamics properties, namely Absorption, Distribution, Metabolism, Excretion and Toxicity (ADMET) of the compounds were calculated using the online server pkCSM (https://biosig.lab.uq.edu.au/pkcsm/). Amphotericin B & miltefosine served as controls to enhance the interpretation^31^.

### Molecular dynamics (MD) simulation

Molecular dynamics simulations were conducted on the *LdmtPRI1* docked complexes using the Desmond software package^49^ by Schrödinger, LLC^50^. The simulations extended for 300 ns (ns) to observe the formational changes in the protein resulting from the protein–ligand complex formation. The aim was to assess the impact of these conformational changes on the protein–ligand complex^51^ under simulated physiological conditions, employing Newton's classical equation of motion^52^. The proteins and ligands were individually pre-processed using Maestro's Protein Preparation Wizard. For simulation, the orthorhombic box was designated as a solvent model; the arrangement was built with the System Builder tool via solvation using TIP3P and OPLS_2005 force field^53^. Appropriate counter ions (Na+/Cl^-^) and a salt concentration (0.15 M NaCl) were utilized to neutralize the solvating system and to imitate physiological circumstances^54^. Throughout the simulation, the NPT (Nose–Hover Thermostat)^55^ maintained a temperature of 300 K and a pressure of 1 atm. Root mean square deviation (RMSD), Root mean square fluctuations (RMSF), Radius of gyration (Rg), Hydrogen-bonds (H-bonds) and Secondary structure elements (SSE) were assessed to ascertain the stability of the protein–ligand complex^56^.

#### Principal component analysis (PCA) and dynamic cross-correlation matrix (DCCM) analysis

This study delved into the flexibility of the *LdmtPRI1* complex by examining the collective movements of the protein–ligand complex. The methodology involved removing translational and rotational motions linked with the proteins. Subsequently, the coordinates were aligned with a reference structure to compute the positional covariance matrix of atomic coordinates and their respective eigenvectors. This symmetric matrix underwent diagonalization using an orthogonal coordinate transformation matrix, resulting in a diagonal matrix showcasing eigenvalues. Each eigenvector in this matrix represented an eigenvalue denoting the total mean-square fluctuation of the system along the specific eigenvectors. The covariance matrix (C) was calculated using the following equation.

$${\text{Cij}} = \left\langle {\left( {{\text{xi}} - \left\langle {{\text{xi}}} \right\rangle } \right)\left( {{\text{xj}} - \left\langle {{\text{xj}}} \right\rangle } \right)} \right\rangle \quad \left( {{\text{i}},{\text{j}} = {1},{2},{3}, \ldots .,{\text{3N}}} \right)$$

where N stands for the count of Cα-atoms, xi/j refers to the Cartesian coordinate of the ith/jth Cα-atom, and <xi/j> signifies the time-averaged value across all conformations. This PCA study was conducted to reconstruct the comprehensive arcs throughout the simulation of 300 ns, calculated by constructing a covariance matrix^57^. The DCCM was constructed through Cα-ATOMs throughout 300 ns MD simulation for *LdmtPRI1* bound complex to explore domain relationships. PCA and DCCM were analyzed using the Bio3D package of R using a script written in R language^58,59^.

#### Molecular mechanics-generalized born surface area (MM-GBSA) calculations

The Python script thermal mmgbsa.pyn within the prime module^60^ was utilized to evaluate the binding free energies (Gbind) of the protein–ligand complex employing the MM-GBSA method. These free binding energies were computed using the OPLS 2005 force field, VSGB solvent model, and rotamer search method^61^. The following equation determines the calculation of the binding free energy upon receptor-ligand binding:

$$\Delta {\text{Gbind}} = {\text{Gcomplex}} - \left( {{\text{Gprotein}} + {\text{Gligand}}} \right)$$

where ΔGbind = binding free energy, Gcomplex = free energy of the complex, Gprotein = free energy of the target protein, and Gligand = free energy of the ligand.

### Parasite inhibition assay

Cytotoxicity of both the test compounds against *L. donovani* promastigote was performed via MTT tetrazolium reduction assay^62^. This colorimetric analysis is based on the reduction of MTT [3-(4, 5-dimethylthiazol-2-yl)-2, 5-diphenyltetrazolium bromide] dye into an insoluble purple colour product, formazan by mitochondrial enzymes in viable cells. The compounds of interest, benfotiamine and capecitabine (Sigma-Aldrich, USA, catalogue number B9636 and SML0653, respectively), were dissolved in 0.1% DMSO and subjected to screening to assess their impact on cellular cytotoxicity^63^. Briefly, promastigote cell culture of *L. donovani* (2 × 10^6^ cells/mL) was dispensed into flat-bottom 96-well clear polystyrene plates (Tarsons, India) at a volume of 100 µl/well for the assay and left to incubate overnight. Following incubation, the test compounds, solubilized in 0.1% dimethylsulfoxide (DMSO) at concentrations (1–40 μM), along with the positive control (Amphotericin B), were added to the promastigote cells. Concurrently, untreated cells were included as the negative control. After this, the cultures were transferred to an environment shielded from light and incubated overnight at 26° C. Post-incubation, cells were pelleted, and the culture medium was removed. These cells were treated with MTT reagent (5 mg/mL) obtained from the In Vitro Toxicology Assay Kit (TOX-1, Sigma-Aldrich, USA)^64^ and incubated in darkness at 26° C for 4 h. Subsequently, the MTT reagent was suctioned, and the MTT solubilizer (100 μl/well) was added to dissolve formazan crystals. The reduction of MTT was quantified by measuring absorbance at 570 nm using a Microplate Reader (Thermo Scientific, USA) to determine cell viability. IC_50_ values for each compound were calculated using GraphPad Prism version 9.0 (http://www.graphpad.com/). The assay was conducted in triplicate, and results from three independent experiments were analyzed.

### Cloning and purification of *LdmtPRI1*

The *LdmtPRI1*gene was PCR amplified from Ld1s genomic DNA using end primers (5′CACCGAATTCCATATGCAGCGTCTTACGTCTGCC3′) and (5′TCGGATCCTCCAGCTCGACGGAACGCC3′) (Hysel, India) and cloned into SmaI site of pUC19 vector (Addgene, USA) and sequenced to ensure authenticity. The recombinant plasmid was cloned into the BamH1 and EcoR1 sites of the pET28a(+) vector (Addgene, USA). The protein was expressed in 1L LB broth (Himedia, India) with *E. coli* BL21 cells induced with 0.5 mM IPTG (Sigma-Aldrich, USA) and incubated for 20 h at 16°C. The expressed histidine-tagged protein was purified with Ni^2+^-NTA agarose (Takara, India) and eluted with buffer (50 mM Tri-Cl, pH 7.5, 300 mM NaCl, and 250 mM Imidazole) (Sigma-Aldrich, USA) and analyzed by Sodium dodecyl-sulphate polyacrylamide gel electrophoresis. The final eluted fraction was concentrated with Pierce™ Protein Concentrator PES, 10 K MWCO (Thermo Scientific) and aliquots were stored with 25% glycerol (V/V) at − 80° C for further studies.

### Primase activity assay

The quantification of purified primase obtained through Ni^2+^-NTA chromatography was conducted using the Bradford assay^65^. Following the method devised by Biswas and colleagues, a coupled primase–pyrophosphatase assay was employed to evaluate and quantify primase activity^66^. Reactions were set up with 40 nM primase (*LdmtPRI1*), 1.25 µM M13mp18 Single-stranded DNA (7249 bp) (New England Biolabs, UK), 100 µM NTP (Takara, India), 50 mM NaCl (Sigma-Aldrich, USA), 150 mM Potassium glutamate (Sigma-Aldrich, USA), 20 mM CAPS (3-(Cyclohexylamino)-1-propanesulfonic acid) buffer pH 8.8 (Sigma-Aldrich, USA), 2 mM Mg^2+^ (Sigma-Aldrich, USA) and 1 U pyrophosphatase (Sigma-Aldrich, USA) in flat-bottom 96-well clear polystyrene plates (Tarsons, India) and maintained at 22° C for 1 h. PPiase selectively cleaves pyrophosphate (PP_i_) into two phosphates (P_i_) and does not hydrolyze nucleotide triphosphates, thus allowing us to inspect PP_i_ release through detection of P_i_^67^. Three volumes of the malachite green reagent (Sigma-Aldrich, USA) were added to the reaction mix and maintained at RT for 5 min, followed by 10% sodium citrate (Sigma-Aldrich, USA) and kept at RT for a minute. Following a 30-min incubation to enable colour development, the absorbance was assessed at 650 nm^66^ using a Microplate Reader (Thermo Scientific). The colorimetric primase–pyrophosphatase assay offers a quantitative assessment of primase activity by detecting the release of PPi during the incorporation of NTP into developing RNA^66^. The experimental results were based on triplicates of the assay performed.

#### Primase inhibition assay

Assorted concentrations (1 nM–1 μM) of compounds (benfotiamine and capecitabine (Sigma-Aldrich, USA) prepared in 0.1% DMSO was used in triplicate for the primase inhibition studies, and the inhibition reaction was set up similarly to that of the primase assay. A negative control of 0.1% DMSO was introduced concerning the drug, whereas an optimized primase reaction (Methods 2.11) was taken as the positive control. The reaction mixture was maintained at 22° C for 1 h. Three volumes of the malachite green reagent (0.0812% malachite green 2.32% w/v polyvinyl alcohol, 5.72% in 6 M of HCl ammonium molybdate and water in the ratio 2:1:1:2 respectively) were added, trailed by 10% sodium citrate to the reaction to develop colour and absorbance was measured at 650 nm. GraphPad prism vs 9.0 (http://www.graphpad.com/) was utilized to calculate the IC_50_ values of enzyme inhibition^66^. The triplicate of the assay was used as an experimental parameter.

### Statistical analysis

All the experiments were conducted autonomously, with each trial replicated at least three times in triplicate. Statistical analyses were performed utilizing the unpaired Student's t-test and executed on GraphPad Prism version 9.0 (http://www.graphpad.com/).

## Results

### De novo *LdmtPRI1* structure prediction, evaluation and energy refinement-

Owing to the unavailability of *LdmtPRI1* crystal structure, we built its predictive model (Figure S1, Supplementary data) using the Robetta server that predicts the 3-dimensional structure of a protein based upon the RoseTTA Fold algorithm^35^ utilizing the amino acid sequence as input (https://robetta.bakerlab.org/).

PROCHECK was deployed to validate the models using Ramachandran's plot. The 4th model was intended for further study as it covered 92.6% of residues in the favoured region, 7.4% in additional allowed regions, and no residues in the generously allowed or disallowed regions of the Ramachandran plot (Figure S2 & Table S1, Supplementary data).

The best model (Model No. 4) was refined via the Energy minimization server YASARA to enhance the model’s stereochemistry. The refined model (Model No. 4) was then analyzed via the ProSa server for validation. The Z-score of *LdmtPRI1*, determined using the ProSa, was found to be – 6.54 (Figure S3, Left, Supplementary data). In the energy plots, the average energy is shown by the thick line, which has a window size of 40 residues; additionally, the thin line within the plot’s background denotes the mean energy about each of the 10 residue fragments (Figure S3, Right, Supplementary data). The energy plot by ProSa signified a decent model for further study.

### Molecular docking

Molecular docking plays an essential and vital function in conceptual drug development and is a powerful and efficient approach for computational screening^68^. Molecular docking studies how ligand molecules align and conform when they bind to target proteins, where actual positions produced by algorithms are ranked using scoring systems^43^. Determining the correct binding mode in which ligands bind within the protein cavity is an arduous task in computational chemistry^69^. Virtual screening based on ligands was used in this study. Virtual screening with a structure-based approach is significant and complements conventional screening methods^70^. The PyRx tool was used in the docking procedure. Based on the results from the docking processes, the top poses with the lowest dock scores were studied further and considered for visual representation. The ligands benfotiamine, capecitabine, febuxostat, rolipram, and varespladib exhibited the best binding energies, as shown in Table 1.

**Table 1:** Binding energy values (docking score) and interactions analysis between the top five ligands with *LdmtPRI1* model in PyRx along with the respective H-bonds and bond lengths.

Ligand	Binding energy (kcal/mol)	Key residues interaction	H-bonds	Bond length (Å)
Varespladib	− 8	ASN451	ND2–O4	2.95
Benfotiamine	− 7.6	ARG148	NH2–O2	2.95
		GLU155	OE2–O4	3.26
		LYS254	NZ–O5	3.09
Rolipram	− 7.5	LYS254	NZ–O2	3.07
			NZ–O3	3.16
Capecitabine	− 7.2	ARG148	NE–O5	3.06
		LYS254	NZ–O1	3.26
Febuxostat	− 7.1	ARG148	NH2–O3	3.14
			NE–O2	2.80
		GLU155	OE3–O3	3.14

**Figure 1:**
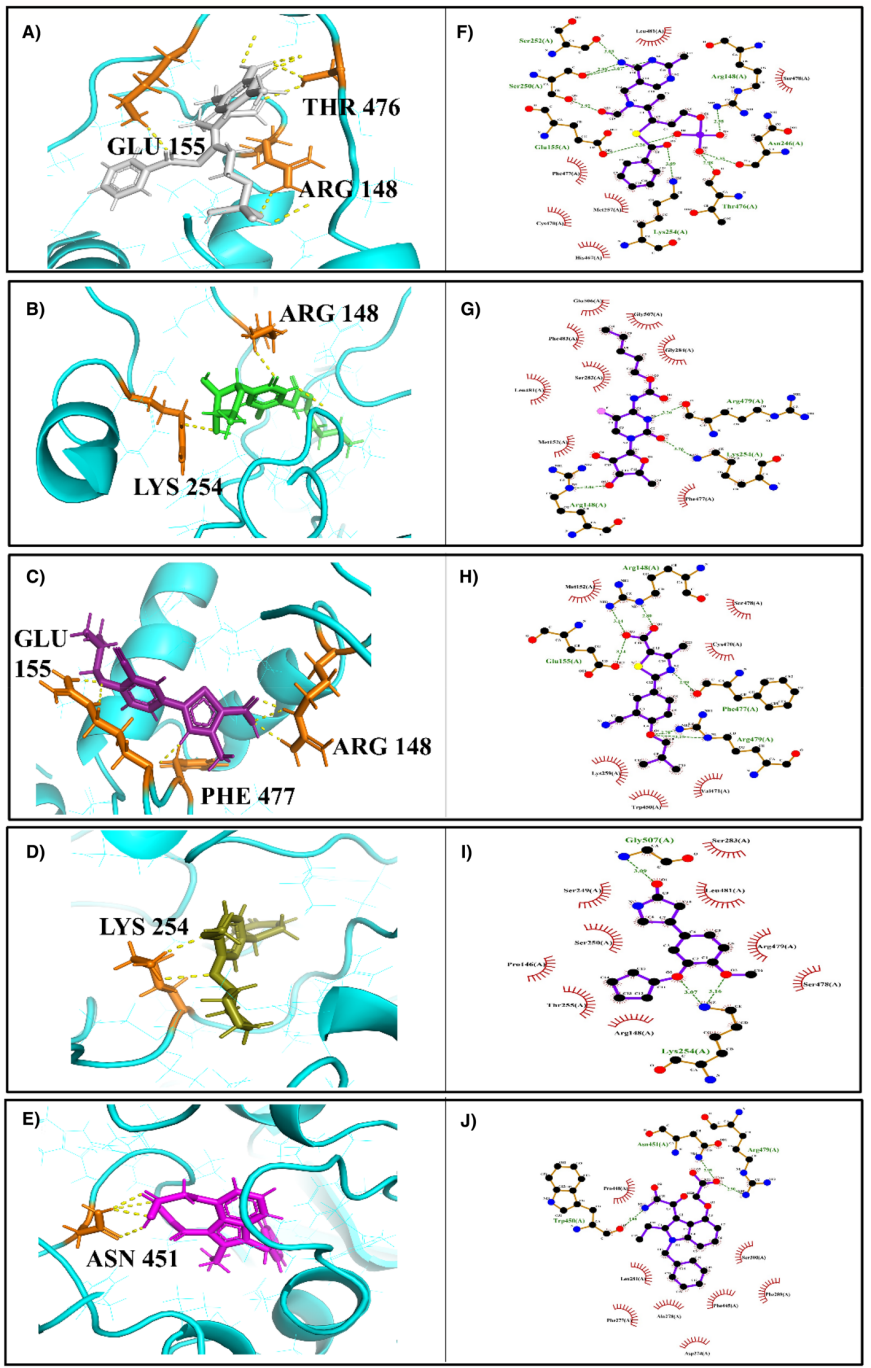
3D & 2D interaction representation of the top five molecules in the active site of the *LdmtPRI1*. Docking poses showing interaction of Benfotiamine (**A**,**F**), Capecitabine (**B**,**G**), Febuxostat (**C**,**H**), Rolipram (**D**,**I**), and Varespladib (**E**,**J**). Hydrogen bonds are represented in yellow (3D) & green (2D).

The top five compounds were analyzed post-molecular docking for their bond lengths, stable hydrogen bonds and interaction with residues within the protein. Active site residues used for docking of *LdmtPRI1* against drug repurposing compounds are presented in Table 2. Pymol software was used to visualize the docked complexes in their three-dimensional configuration (Fig. 1A–E), whereas the two-dimensional interactions were observed with Ligplot (Fig. 1F–J). The ligand benfotiamine (dock score: − 7.6 kcal/mol) interacted and forged 4 stable H-bonds with the binding site residues, ARG 148, GLU 155, LYS 254 and THR 476 of the target protein (*LdmtPRI1*) (Fig. 1F), capecitabine (dock score: − 7.2 kcal/mol) form 2 stable H-bonds with ARG 148 and LYS 254 residues of the target protein (Fig. 1G), the ligand febuxostat (dock score: − 7.1 kcal/mol) interacted with the protein of interest in the residues ARG 148, GLU 155 and PHE 477 establishing four stable hydrogen bond (Fig. 1H), rolipram (dock score: − 7.5 kcal/mol) formed two hydrogen bonds at residue LYS 254 with target protein (Fig. 1I) and varespladib (dock score: − 8 kcal/mol) had only one stable hydrogen bond at residue ASN 451 of *LdmtPRI1* (Fig. 1J).

**Table 2:** Active sites residues of *LdmtPRI1* for docking against drug repurposing compounds.

Residue number	Residue
148	Arginine
155	Glutamic acid
246	Asparagine
254	Lysine
259	Lysine
266	Valine
451	Asparagine
470	Cysteine
476	Threonine
477	Phenylalanine
481	Leucine

As DNA primase requires nucleotides and single-stranded DNA as substrates for replication initiation, we also docked ATP and DCP (positive controls) against *LdmtPRI1* to determine its substrate binding sites. Blind docking and interaction analysis exhibited docking scores of − 7.8 and − 6.4 kcal/mol for ATP and DCP, respectively, signifying substantial interaction and a pronounced affinity towards *LdmtPRI1*. Further 2D and 3D interaction analysis revealed DCP and ATP formed H-bonds with residues ARG 148 and THR 476 (Supplementary Figure S4A–D), thereby suggesting that these residues are also probable substrate binding sites in *LdmtPRI1* other than active sites for ligand binding. Thus, selected drugs might also utilize the protein's substrate binding site to inhibit DNA replication.

*LdmtPRI1’s* in-silico dead variant was generated via a computational method. The amino acid residues ARG 148, GLU 155, ASN 246, LYS 254, LYS 259, VAL 266, ASN 451, CYS 470, THR 476, PHE 477, and LEU 481 in the substrate binding site were substituted with Alanine residues. The three-dimensional conformation of the in-silico mutant was predicted utilizing the Robetta web server, and the model's stereochemical integrity was assessed using PROCHECK^71^. A docking score of − 1.6 kcal/mol between the mutant protein and substrate ATP was found, suggesting a lowered affinity for the substrate binding site. The docked complex’s 3D and 2D analysis showed that mutations in the substrate binding site residues of the protein fail to establish any H-bonds with the substrate molecule. An in-silico study of the substrate binding residues revealed the probable amino acids responsible for substrate binding. Mutations in these residues can significantly decrease the protein's activity Supplementary Figure S5.

### Drug likeness properties of ligands

The drug-likeness parameters and pharmacokinetic profiles were investigated where all ligands met Lipinski's rule of 5, indicating high permeability. All the ligands have molecular weights less than 500 Daltons and water/octanal partition coefficient (log P) values less than 5, indicating that the ligands are cell membrane permeable^72^. The ligand's drug-likeness scores were calculated via Molsoft L.L.C.: Drug-Likeness software and no ligands exhibited negative drug scores. The results of Lipinski's parameters and drug-likeness scores are listed in Table S2, Supplementary data.

### ADMET profiling

Pharmacokinetic properties, i.e. (ADMET) profiles of the selected compounds, were analyzed via pkCSM software. Amphotericin B and miltefosine were taken as a control for better interpretations. The compounds are seen to be optimally water-soluble in terms of absorption, hence displaying an improved absorption rate in the human intestinal mucosa. The skin permeability of most of the compounds is positive, and they are non-inhibitors of human P-glycoprotein, suggesting that the compounds are uniformly adsorbed throughout the cell membrane (Table S3, Supplementary data). The tested compounds can be projected as ideal drug candidates (Table S3, Supplementary data) as they have been distributed efficiently over the cell membrane and are pervious to the brain barrier and central nervous system. The compounds also represent excellent metabolism (Table S3, Supplementary data) as they are non-substrate based and non-inhibitors of cytochrome p450. The total renal clearance (CL_tot_) values of the compounds are moderate, whereas the values of the human Organic cation transporter 2 (OCT2) substrate (Table S3, Supplementary data) were observed as negative. Apart from being non-inhibitors of human ether-a-go-go related gene (hERG) I & II, the compounds displayed negative potential towards Ames toxicity. These compounds did not induce skin sensitization and displayed a decent level of Oral rat chronic toxicity (LOAEL) (Table S3, Supplementary data).

### MD simulation study

**Figure 2:**
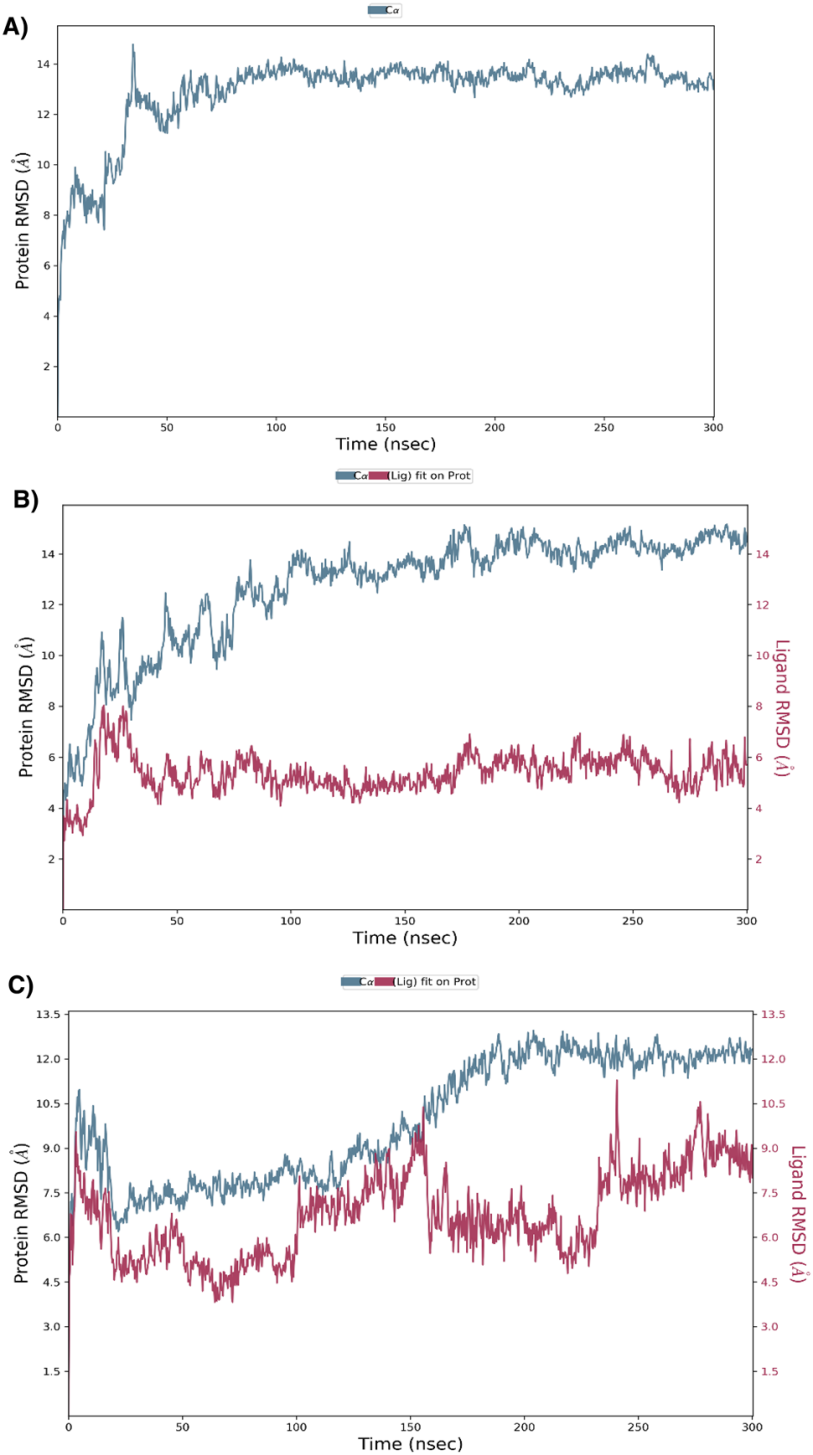
The root mean square deviation (RMSD) between the C-alpha atoms of *LdmtPRI1* and ligands over time. (**A**) RMSD plot of *LdmtPRI1* (protein-only), (**B**) RMSD plot of benfotiamine bounded with *LdmtPRI1* and (**C**) RMSD plot of capecitabine bonded with *LdmtPRI1*. Differences in ligand root-mean-square deviation (RMSD) over time are presented on the right Y-axis.

Molecular Dynamics Simulation tests were performed to evaluate the equipoise of ligands bound to *LdmtPRI1* protein using Schrodinger Suite’s Desmond Simulation software to corroborate the conformational stability of the protein–ligand complexes. Backbone RMSDs were analyzed throughout a 300 ns simulation duration to determine the binding site stability of the compounds based on their projected affinity towards the binding site of *LdmtPRI1*. RMSD analysis was performed by assessing the Cα atoms of the *LdmtPRI1* protein, which were then compared to the simulation time. RMSD plot for *LdmtPRI1*, as illustrated in Fig. 2A, reveals significant instability until 50 ns with deviations greater than 14 Å but attained stability post 100 ns and maintained stability throughout the simulation period. The obtained RMSD trajectories for the *LdmtPRI1*, with respect to its C-α backbone, rises with the RMSD > 14 Å at 40 ns but gradually decreases and was stable from (50–300) ns throughout (Fig. 2A). High RMSD values can affect the accuracy of predicting the binding site and interactions between the protein and ligand^73^. Moreover several study supports that functional regions of a protein often exhibit higher flexibility, leading to higher Root Mean Square Deviation (RMSD). Protein flexibility is frequently important for their ability to adapt to different biological activities. These dynamic character of the functional regions may be shown by the variation in backbone structure, as determined by RMSD^73–75^. Additionally, RMSD is just one measure of structural accuracy, and conjunction with other metrics for a comprehensive evaluation of a protein model^73^. The RMSD plot for benfotiamine confined to *LdmtPRI1*, as presented in Fig. 2B, conceals that in the initial 30 ns, the ligand-bound protein exhibited substantial stability, although it was marginally uneven within the period of 50–200 ns; conversely, the complexes stabilized after 200 ns simulation. On the contrary, the RMSD plot of capecitabine merged with *LdmtPRI1* is presented in Fig. 2C, where between 30 and 120 ns simulation period, stabilization was achieved by ligand-binding complex. The complex displayed variability from 150 to 200 ns simulation time, although a slender deviation was witnessed in the complexes, which stabilized after 200 ns up to 300 ns. The validation of interaction stability between the ligand and protein is confirmed when the RMSD value of the backbone remains below 2.5 Å^76^. Protein–ligand exhibited a subtle deviation in RMSD, as evidenced by the backbone RMSD analysis. The observed deviance is likely attributed to conformational changes occurring in the rotatable bonds of the ligand, as evidenced by the presence of these bonds in the two-dimensional representation (Fig. 1F–J) of the protein–ligand interactions. These deviations arise from fluctuations in the torsion angles of the ligand^77^.

**Figure 3:**
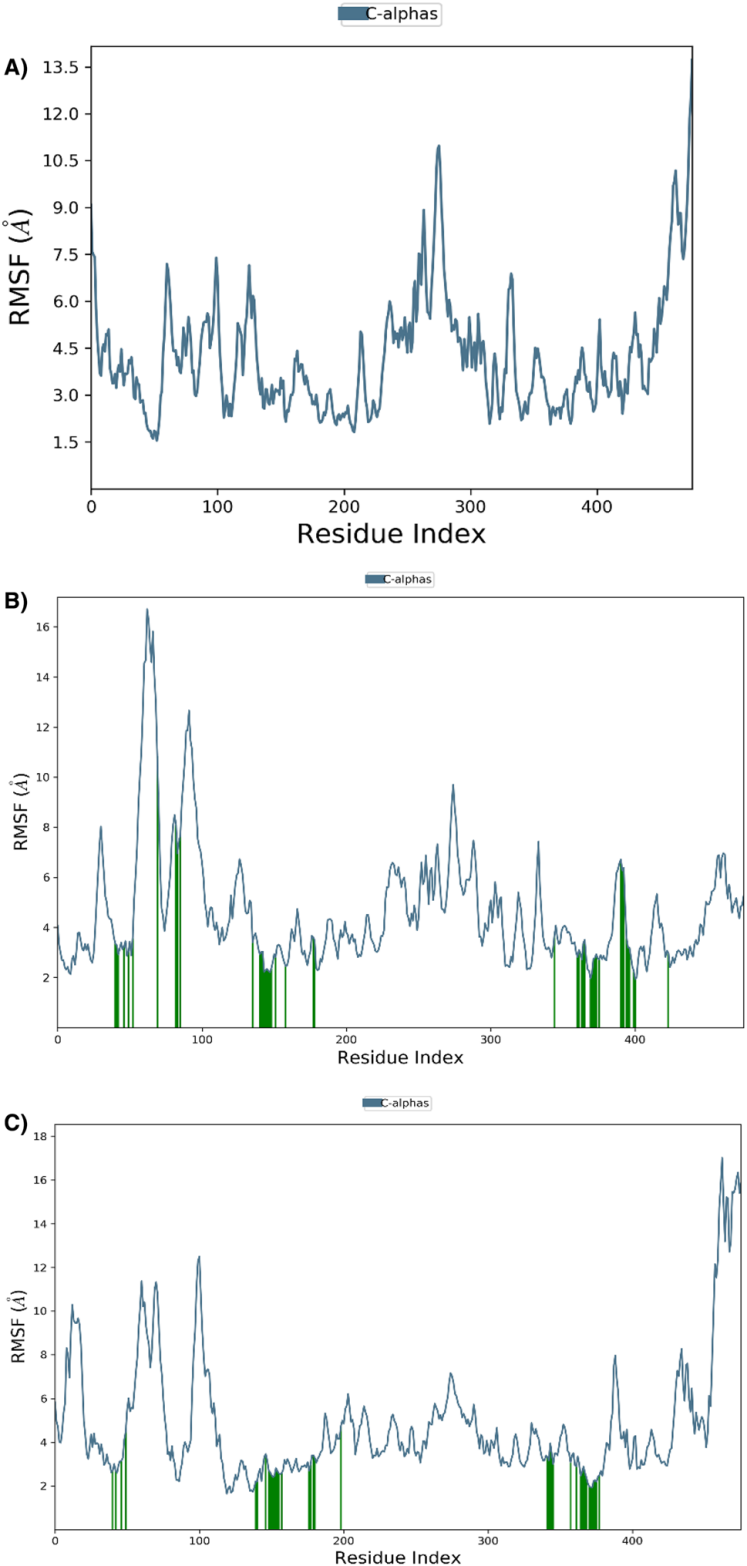
RMSF plot of *LdmtPRI1* residues complexed with the selected ligands. (**A**) Plot of root mean square fluctuations (RMSF) of *LdmtPRI1*(protein-only), (**B**) RMSF plot for benfotiamine bound *LdmtPRI1,* (**C**) Plot of RMSF values of capecitabine bonded to *LdmtPRI1*.

The variation in individual amino acid residues and the degree of displacement or alteration of those residues during a simulation run is evaluated via RMSF. Minimal fluctuations in the atoms at the active site and the main chain suggest minimal conformational change, suggesting that the claimed top compound is well-maintained within the protein binding pocket cavity^78^. RMSF plot of the ligand–protein complexes indicated minimal fluctuations of all complexes that did not change significantly during the 300-ns simulation period and remained consistent across all complexes, as illustrated in Fig. 3A–C.

**Figure 4:**
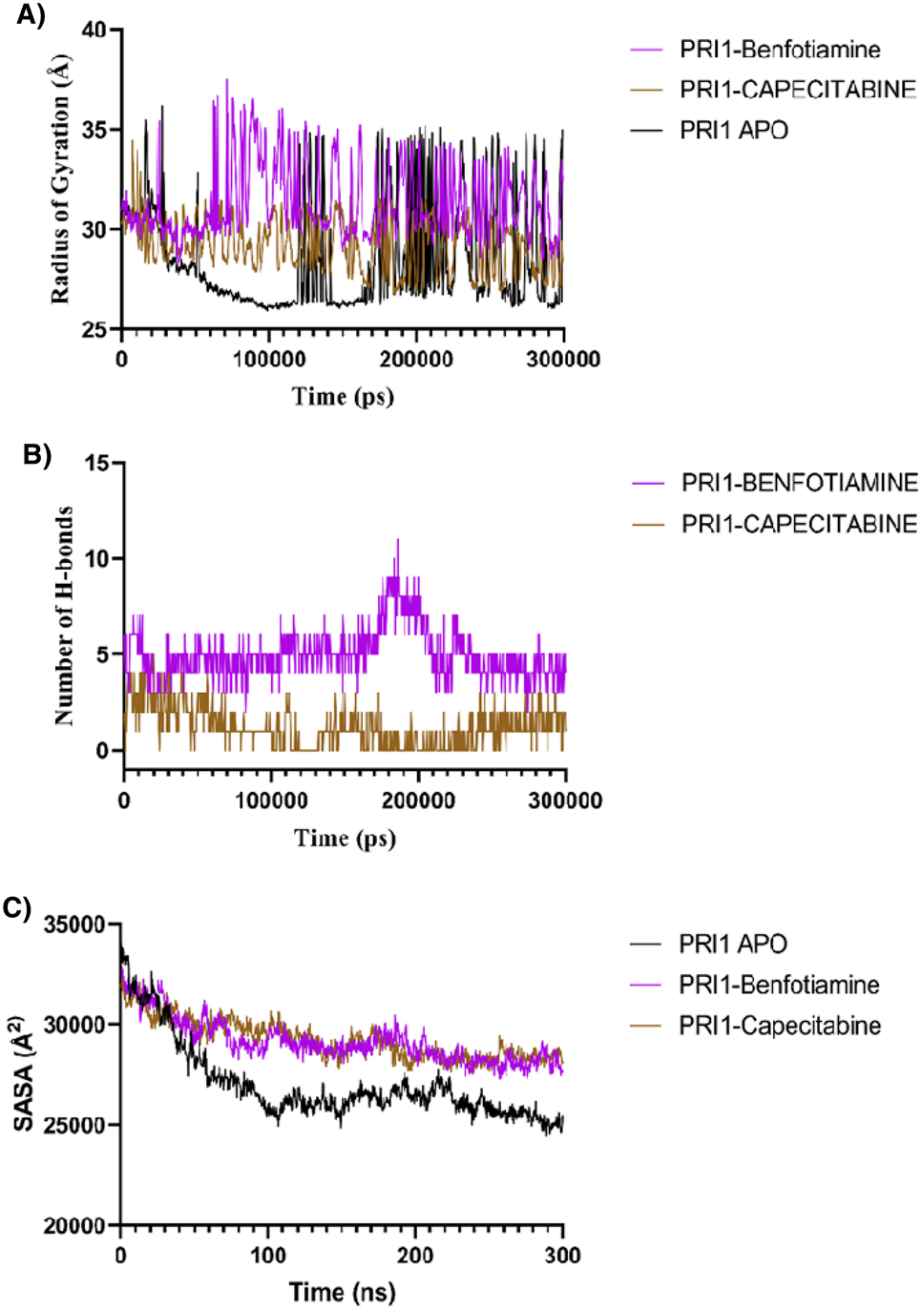
(**A**) Time progression graph of radius of gyration (Rg) during simulation *LdmtPRI1* (black), *LdmtPRI1*-benfotiamine (purple) and *LdmtPRI1*-capecitabine (brown). (**B**) H-bonds within the protein–ligand complexes generated during simulation, *LdmtPRI1*-benfotiamine (purple) and *LdmtPRI1*-capecitabine (brown). (**C**) Solvent-accessible surface area (SASA) results of *LdmtPRI1* (black) *LdmtPRI1*-benfotiamine (purple)& *LdmtPRI1*-capecitabine (brown).

To investigate the conformational properties of the protein–ligand complex, we calculated the Rg plots associated with the protein's structural firmness (Fig. 4A). Results indicate that the Rg trajectories of *LdmtPRI1* attain equilibrium during the early 20 ns of ∼ 31 Å and stay steady with negligible perturbation at 50–100 ns. In contrast, the arc of *LdmtPRI1* complexed with benfotiamine achieved an equilibrium of 29–32 Å during the initial 0–40 ns, but the complex demonstrates sharp drifts of R_g_ approximately 29–32 Å during the ∼ 75–300 ns. The complex *LdmtPRI1*-capecitabine was perceived to remain stable around 0–20 ns but have marginal perturbation in radius of gyration with approximately 27–31 Å during the ∼ 50–300 ns period.

The H-bonding interactions are vital for the molecular integrity of protein configuration and for ensuring the stable spatial localization of ligands within the protein’s active site. The occupancy of H-bonds by ligands, benfotiamine and capecitabine with *LdmtPRI1* is illustrated in Fig. 4B. Benfotiamine is particularly effective against *LdmtPRI1* and can establish more than ten H-bonds during the 300 ns simulation run with active site residues at ARG 148, GLU 155 and THR 479. At the same time, capecitabine maintained at least 1 H-bond in the active sites ARG 148 and LYS 254 throughout the 300 ns simulation period. Thus, benfotiamine was expected to be more efficient than capecitabine for probable inhibition of *LdmtPRI1*.

A solvent accessible surface area (SASA) study was also performed during a 300 ns MD simulation that quantifies interfaces of the protein–ligand complex with solvents^79^. Throughout the MD run, SASA for *LdmtPRI1* alone was observed to range between 25,000–27,000 Å^2^, the complex *LdmtPRI1*-benfotiamine revealed an SASA value of 27,000–31,000 Å^2^, whereas *LdmtPRI1*-capecitabine had a SASA value of 28,000–32,000 Å^2^ (Fig. 4C). Protein–ligand complexes with high and relatively stable SASA values indicate that the ligand is available for solvents to interact with, devoid of any alterations in the protein structure^80^.

**Figure 5:**
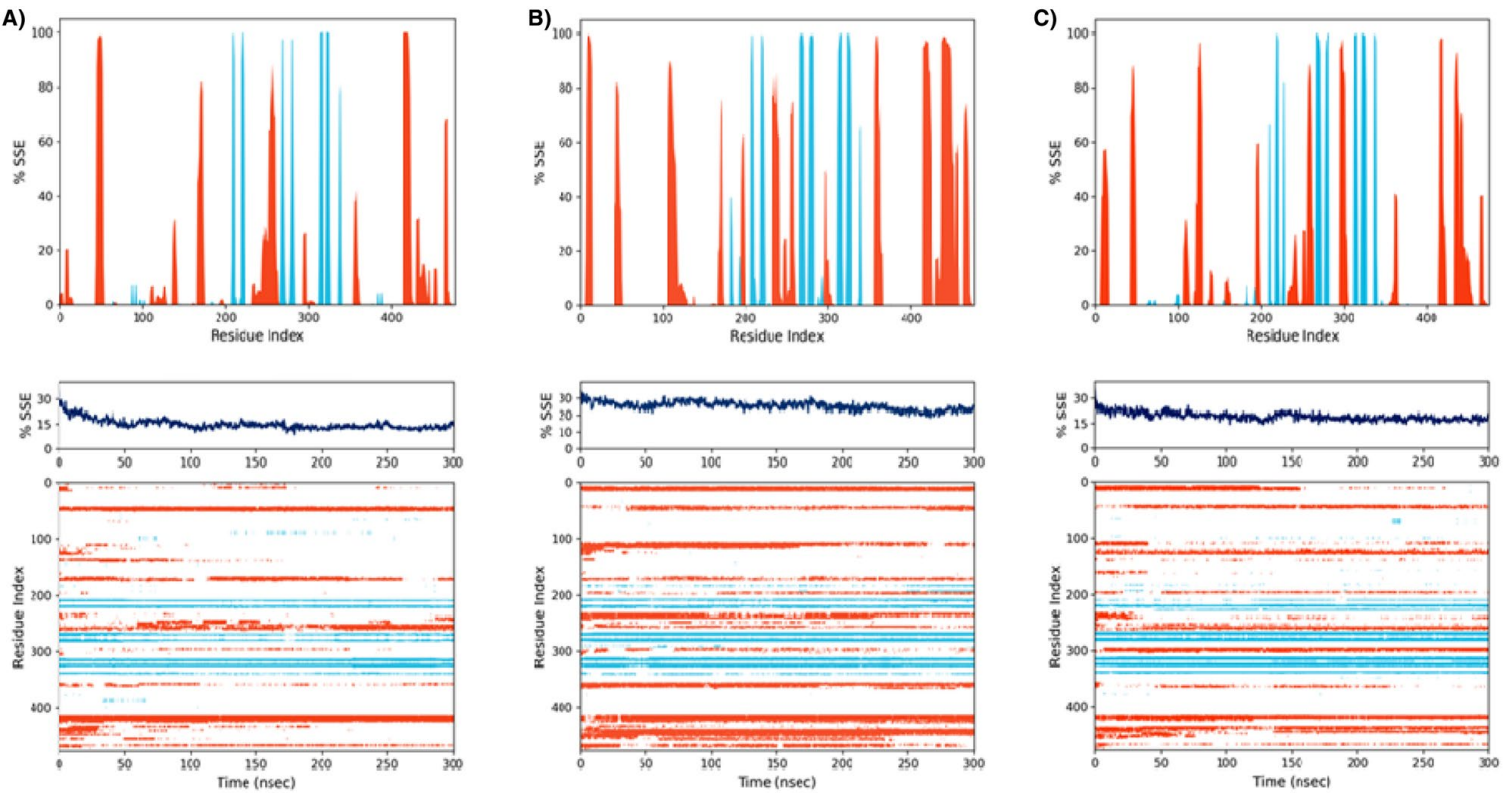
Elements of (**A**) *LdmtPRI1*(protein-only), (**B**) *LdmtPRI1*-benfotiamine and (**C**) *LdmtPRI1*-capecitabine, are distributed across protein–ligand complexes in relation to the residue index. The alpha helices are indicated by red columns, whereas the beta strands are represented by blue columns. The plot above (Protein-SSE Histogram) illustrates SSE distribution based upon residue index across the protein structure.

The secondary structural features, such as alpha-helices and beta-strands, were quantified during the simulation. The residual index was plotted contrary to the secondary structure elements graph to determine the distribution within the protein structure. *LdmtPRI1* is composed of 9.67% helix and 5.33% strand, amounting to 15.00% total SSE (Fig. 5A) as compared to 17.79% alpha helix, 7.52% beta strand constituting 25.31% of total SSE in benfotiamine (Fig. 5B) whereas 11.6% alpha helices, 7.53% of beta strands leading to 19.21% of secondary structures elements (Fig. 5C) were observed with capecitabine. The proportion of alpha helix to beta-strand, too, impacts protein RMSD. Since protein structures comprise rigid regions, the RMSD of residues in these structures was significantly lower than that of the coils and loop residues^81^.

#### PCA and DCCM analysis

**Figure 6:**
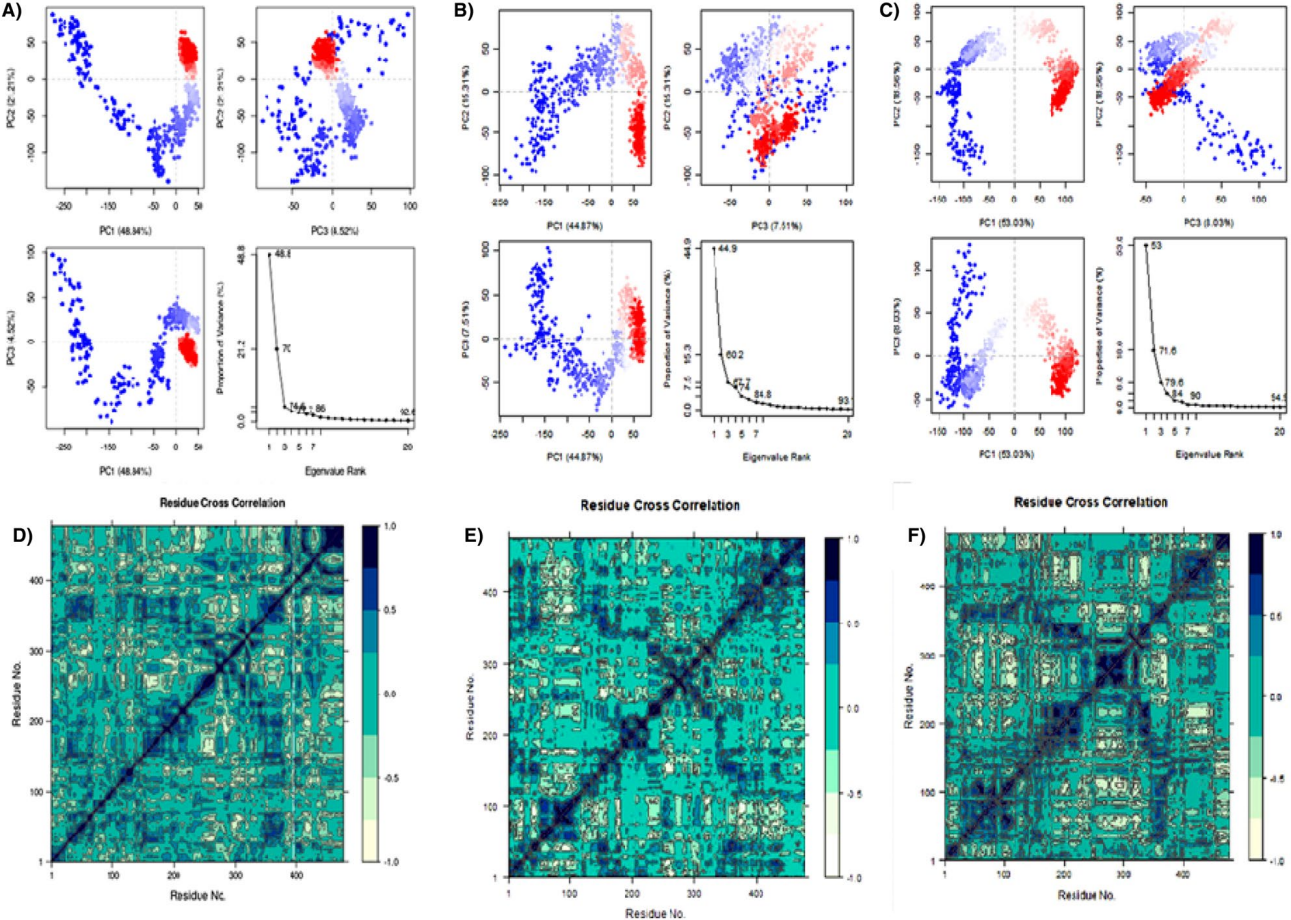
Principal Component Analysis (PCA) & eigenvalue mapped versus the percentage of variance for 300 ns simulation trajectories: (**A**) *LdmtPRI1* only (Variations in PC1, PC2, and PC3 add up to 48.84%, 21.21% and 4.52%), (**B**) *LdmtPRI1*-benfotiamine (Variations in PC1, PC2, and PC3 add up to 44.87%, 15.31% and 7.51%), (**C**) *LdmtPRI1*- capacitabine (Variations in PC1, PC2, and PC3 add up to 44.87%, 15.31% and 7.51%). Dynamic cross correlation matrix (DCCM) plots for (**D**) *LdmtPRI1*-protein only (**E**) *LdmtPRI1*-benfotiamine, (**F**) *LdmtPRI1*- capecitabine. The positive correlated motions are represented in dark blue, negative anti-correlated motions are represented in white and mixed correlation are represented in cyan.

Random global mobility of amino acid residue’s atoms was interpreted using principal component analysis via MD simulation trajectories for ligands bound to *LdmtPRI1*. PCA evaluates the flexible dispersed trajectories caused by protein structural deformation. The movement of the internal coordinates into three dimensions during a spatial duration of 300 ns is documented in a covariance matrix. In contrast, orthogonal sets or eigenvectors infer coherent movement of individual trajectories. The conformational sampling was performed for *LdmtPRI1* both independently and in the presence of benfotiamine and capecitabine, and the PC1, PC2 and PC3 projections were established using the Cα-atoms (Fig. 6A). Each blue, white and red dot represents maximum, intermediate, and reduced motility, respectively. The first three eigenvalues of *LdmtPRI1*, whether on its own or in the presence of benfotiamine and capecitabine, accounted for 74.6%, 67.7%, and 79.6% of the conformational variances, respectively. When *LdmtPRI1* was without any ligand, its conformational space stretched from − 250 to + 50 along PC1 (48.84%), − 150 to + 100 along PC2 (21.21%), and − 100 to + 100 along PC3 (4.52%). Notably, the presence of benfotiamine and capecitabine induced changes in the flexibility of *LdmtPRI1*, which is evident in the PCA plot. *LdmtPRI1* with benfotiamine occupied a subspace ranging from − 250 to + 50 along PC1 (44.87%), − 100 to + 100 along PC2 (15.31%), and − 100 to + 100 along PC3 (7.51%) (Fig. 6B); On the other hand, *LdmtPRI1* with capecitabine occupied a subspace ranging from − 150 to + 100 along PC1 (53.03%), − 200 to + 100 along PC2 (18.56%), and − 50 to + 150 along PC3 (8.03%) (Fig. 6C).

DCCM plot was created to investigate the correlated movement of structural domains to achieve a steady conformational state of the complex following the binding to *LdmtPRI1* (Fig. 6D–F). The varied colour patterns of the matrix plot correspond to different degrees of correlation. The positively correlated motions are represented in dark blue, negative anti-correlated motions are represented in white, and mixed correlations are represented in cyan.

#### MM-GBSA Calculations

The MM-GBSA method extensively evaluates the free binding energy between ligands and protein molecules^82^. It was employed here to assess the binding free energy within the *LdmtPRI1* complex when bound to a ligand and examine additional non-bonded interaction energies. The binding energy of the ligands benfotiamine and capecitabine to *LdmtPRI1* is − 69.79 kcal/mol and − 39.39 kcal/mol, respectively (Table 3). ΔGbind is governed by non-bonded interactions such as ΔG_bind__Coulomb, ΔG_bind__Packing, ΔG_bind__H_bond_, ΔG_bind__Lipo, and ΔG_bind__vdW (Table 3).

**Table 3:** Average MM-GBSA binding energy calculation of *LdmtPRI1*-benfotiamine and *LdmtPRI1*-capecitabine obtained from MD Simulation trajectories.

Energies (kcal/mol)	PRI1-benfotiamine	PRI1-capecitabine
ΔdG_bind	− 69.79 ± 11.49	− 39.39 ± 8.21
ΔdG_bind_Coulomb	19.24 ± 4.91	39.39 ± 5.78
ΔdG_bind_Covalent	2.48 ± 0.26	3.18 ± 0.35
ΔdG_bind_Hbond	− 7.76 ± 0.006	− 1.86 ± 0.002
ΔdG_bind_Lipo	− 24.69 ± 1.74	− 26.35 ± 1.96
ΔdG_bind_Packing	− 0.05 ± 0.001	− 0.00 ± 0.00
ΔdG_bind_vdW	− 51.16 ± 1.89	− 48.16 ± 1.54

### Parasite inhibition assay

**Figure 7:**
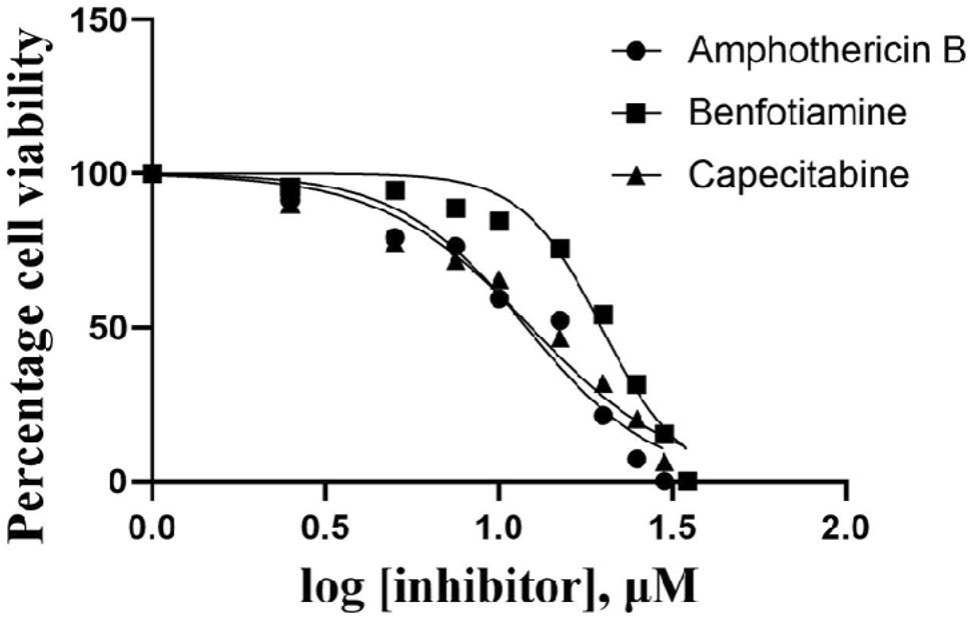
Dose–Response inhibition plot of Amphotericin B (positive control) IC_50_ 12.02  ± 0.09 µM, Benfotiamine IC_50_ 19.79 ± 0.67 µM & Capecitabine IC_50_ 12.40 ± 0.35 µM against *Leishmania donovani* promastigote.

The top two compounds (benfotiamine and capecitabine), which showed good ADMET and pharmacokinetic profiles, were selected for parasite inhibition by performing an MTT tetrazolium reduction assay^62^ to comprehend the effects of the benfotiamine and capecitabine on parasite growth. Drug concentrations (1 μM–40 μM) were prepared in 0.1% DMSO and added to the promastigote culture with an overnight incubation at 26° C. The cellular metabolic activity of the promastigote cells in the presence of drugs was assessed using the MTT assay. Amphotericin B was taken as a positive control drug to interpret the results better. Cell viability was quantified by checking absorbance at 570 nm and IC_50_ (Half maximum inhibitory concentration) values calculated using GraphPad Prism (http://www.graphpad.com/) from inhibition assay, where we observed IC_50_ values of benfotiamine as 19.79 ± 0.67 μM (R^2^ = 0.97; *p* = 0.0162) and capecitabine 12.40 ± 0.35 μM (R^2^ = 0. 97; *p* = 0.0003), in comparison to Amphotericin B with an IC_50_ value of 12.02 ± 0.09 μM (R^2^ = 0.96; *p* = 0.0132) (Fig. 7). These results validate the excellent repressive action of the chosen compounds against the parasite's promastigote form, where we observed that with a logarithmic increase in the concentration of the drugs, there was a substantial reduction in the number of viable cells, indicating inhibitory effects of the compounds in growth and proliferation of promastigote cell culture of *L. donovani*.

### Purification of *LdmtPRI1*

Recombinant plasmid pET28a-*LdmtPRI1* was transformed into *E. coli* BL21 cells and recombinant primase were expressed using 0.5 mM IPTG at 16 °C for 20 h. Cell harvesting was performed via centrifugation, followed by resuspension in lysis buffer (50 mM Tris-Cl, pH 7.5, 300 mM NaCl, 1 × protease inhibitor and 1 mg/mL lysozyme), and sonication was performed. The crude extract was centrifuged, and recombinant *LdmtPRI1* were purified via Ni^2+^-NTA chromatography with buffer (50 mM Tris-Cl, pH 7.5, 300 mM NaCl, 250 mM Imidazole). SDS-PAGE was performed to detect the purified recombinant *LdmtPRI1* protein (Supplementary Figure S6). The eluted fraction was concentrated, and aliquots were stored with 25% glycerol (V/V) at − 80 °C for further experiments.

### Primase activity assay

**Figure 8:**
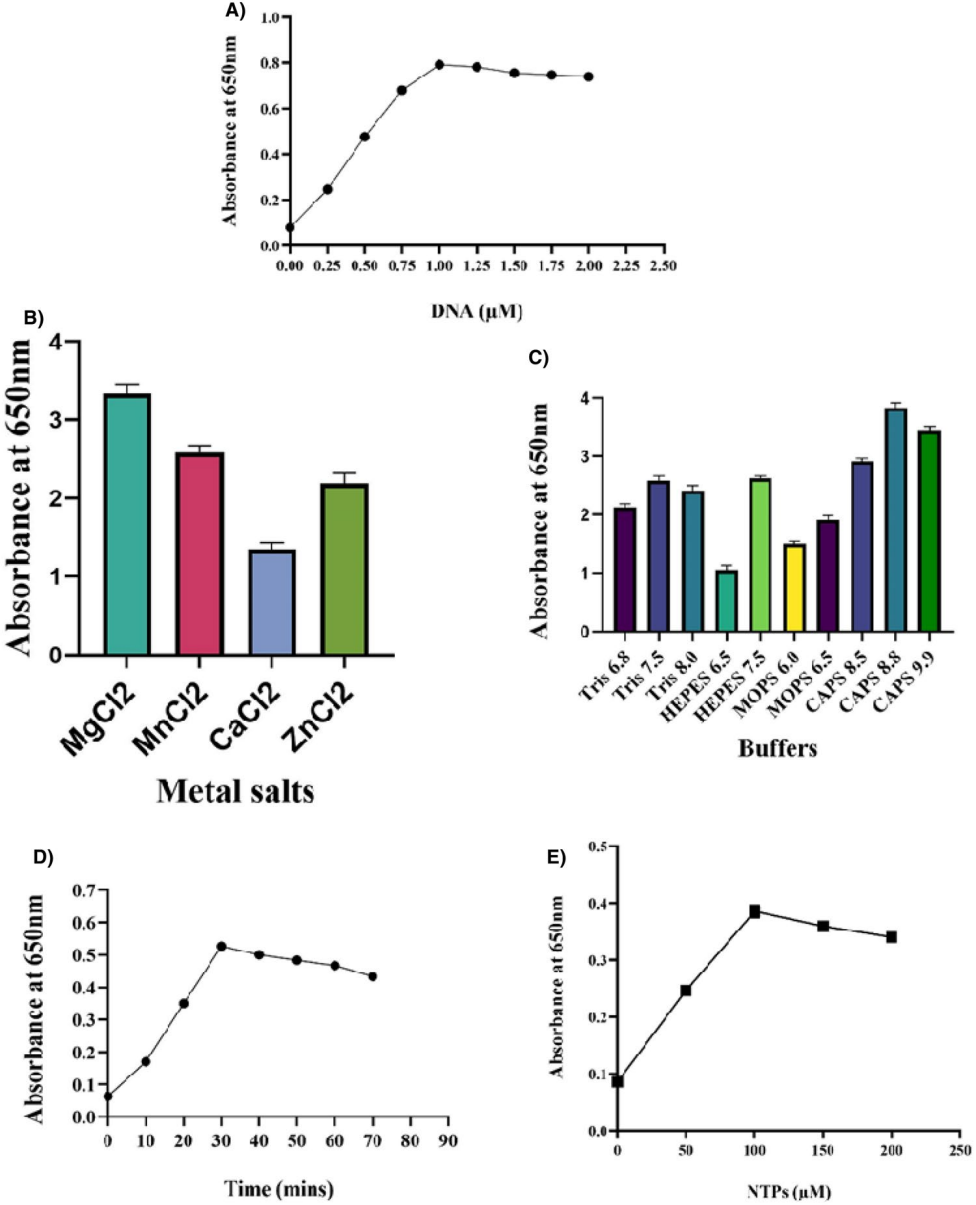
Optimization of parameters for the primase-pyrophosphatase assay. (**A**) Activity of primase at varied DNA concentrations. (**B**) Primase activity assessed in presence of divalent metals salts at 2 mM concentration. (**C**) Primase activity with buffers at pH range 6–10. (**D**) Time course representation of the priming reaction over period of 0-70 min. (**E**) Rate PPi released by primase as a function of NTP concentrations. The experiments were conducted over a duration of 30 min.

With the ELISA plate reader set at 650 nm, the activity of the primase was identified through the detection of Pi (monophosphate) in the coupled primase-pyrophosphate assay where NTPs are utilized by primase to generate short stretches of oligonucleotide upon template releasing PPi (pyrophosphate). Released PPi are converted to Pi, leading to a light blue colour development upon adding MGR to the reaction. The oligo-synthesis activity of primase is directly signified by the presence of Pi during the primase-pyrophosphate assay. A significant primase activity was observed as the optimum extension of template DNA with 1.25uM M13mp18 single-stranded DNA was utilized during the assay (Fig. 8A). Maximal oligo-synthesis by primase is achieved upon adding 2 mM MgCl_2_ salts (Fig. 8B), whereas buffer at pH 8–10 is significant for primase activity (Fig. 8C), as observed during the experiments. The time of less than an hour (Fig. 8D) is viable for priming, as a reduction in the activity of the enzyme was witnessed with the more extended period; increased concentration of NTP (> 100 µM) does not significantly enhance the oligo-synthesis by primase as observed in due course of the primase assay (Fig. 8E). The assays were performed with 40 nM of *LdmtPRI1* protein, and a Microplate Reader measured absorbance at 650 nm^66^.

### Primase inhibition assay

**Figure 9:**
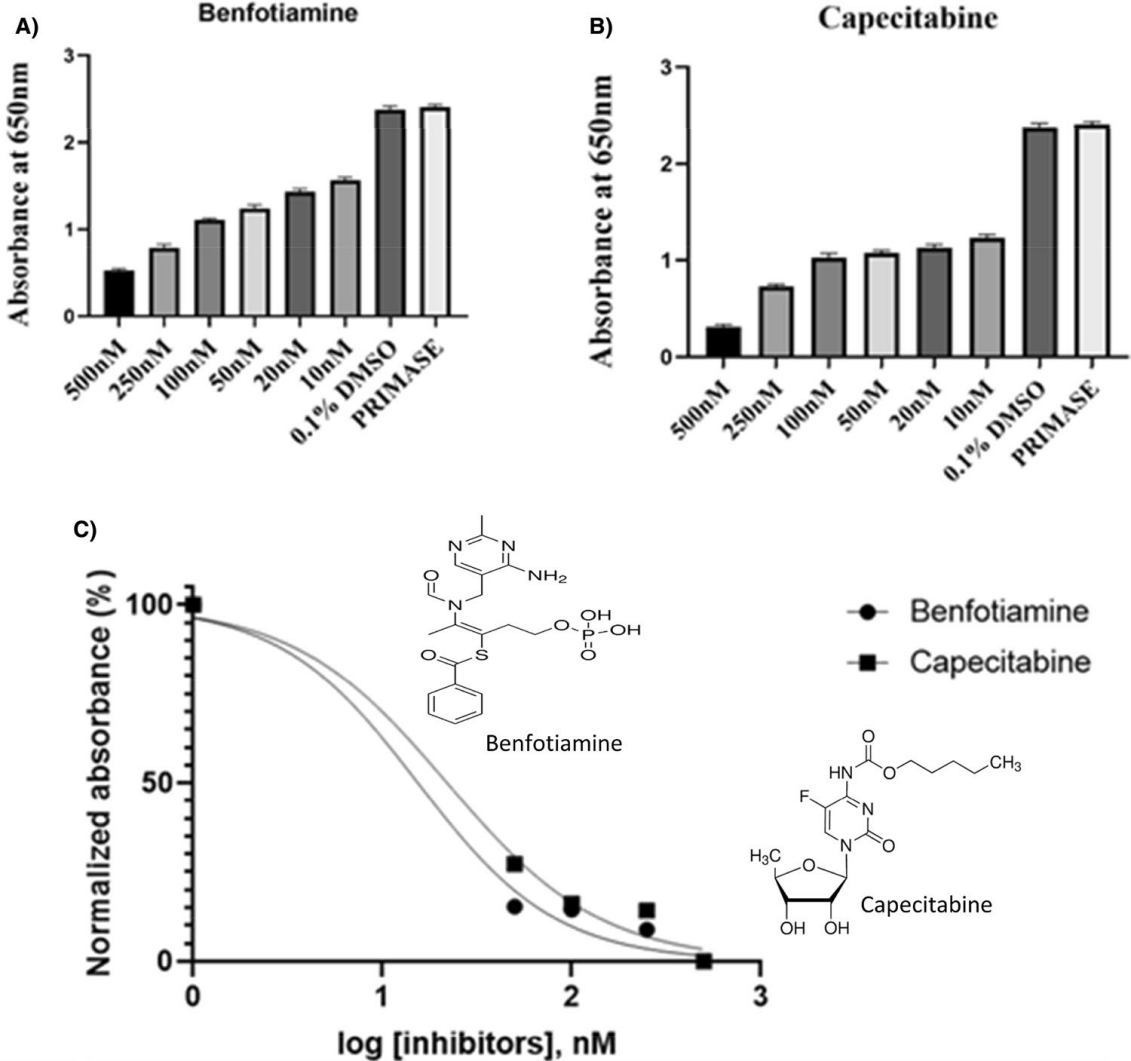
Primase inhibition assay performed with the selected compounds. Bar diagram signifying inhibition of *LdmtPRI1* by (**A**) Benfotiamine and (**B**) Capecitabine. (**C**) Dose response inhibition plot of *LdmtPRI1* by benfotiamine (IC_50_ 20.68 ± 0.03 nM), Capecitabine (IC_50_ 15.27 ± 0.03 nM).

Primase inhibition reaction was set up similarly to primase assay, evaluating the inhibition effects of the selected compounds (benfotiamine and capecitabine) with different concentrations ranging from 10 to 500 nM for the inhibition reactions. For their inhibitory effects on primase's oligo-synthesis capability, selected drugs benfotiamine and capecitabine were studied. The experiments were conducted using 1.25 µM of M13mp18 Single-stranded DNA, 100 µM of NTP and 40 nM of primase (*LdmtPRI1*), and absorbance was measured at 650 nm with a Microplate Reader. IC_50_ values of the selected compounds calculated via GraphPad Prism signifying a logarithmic rise in drug concentration resulted in a noteworthy decrease in oligo-synthesis activity by *LdmtPRI1*, indicating substantial inhibitory effects of the drugs (˂ 25 nM) employed to impede the activity of the *LdmtPRI1* enzyme (Fig. 9A–C). The IC_50_ values calculated using GraphPad prism were predicted to be benfotiamine as 20.68 ± 0.03 nM (R^2^ = 0.98, i = 0.1179) and capecitabine as 15.27 ± 0.03 nM (R^2^ = 0.98, *p* = 0.2939), respectively.

## Discussion

This study chose *Leishmania* kinetoplast (mitochondrial) DNA primase, essential for mitochondrial DNA replication, as a therapeutic target. Primase initiates DNA replication by synthesizing short oligo ribonucleotides on single-stranded DNA templates in the leading and lagging strand known as the Okazaki fragment^20^. The *Leishmania* genome has a unique kinetoplast DNA within the mitochondrion^22^. A kinetoplast mitochondrial DNA primase is necessary to create a mitochondrial DNA replication system in vitro because none of the known DNA polymerases can initiate a DNA strand^23^.

Here, we used drug repurposing approaches to identify the most effective therapeutics for combating Leishmaniasis caused by *Leishmania donovani* by targeting *LdmtPRI1*. Based on virtual drug screening, molecular docking, and MD simulation analysis, the study highlighted the possible inhibitory effect of benfotiamine and capecitabine. Further, the computational findings are validated by parasite inhibition assay and primase activity inhibition assay.

In this current investigation, we performed docking studies of 4240 approved and clinical drug compounds by PyRx utilizing the Autodock Vina docking platform^46^. The results revealed 5 compounds, namely benfotiamine, capecitabine, febuxostat, rolipram and varespladib, possessing the best binding affinity against *LdmtPRI1*. Amongst the top-scored docked compounds, benfotiamine (− 7.6 kcal/mol) formed 4 H-bond with the predicted active site residues ARG 148, GLU 155, LYS 254 and THR 476 of the target protein (Fig. 1F) followed by capecitabine (− 7.2 kcal/mol) with ARG 148 and LYS 254 residues (Fig. 1G) indicating possible top 2 inhibitors of *LdmtPRI1*. Desmond MD simulations were run for 300 ns to obtain information about the structural stability of the five best-docked compounds against *LdmtPRI1* and the protein's apo form. The structure of *LdmtPRI1*-benfotiamine and *LdmtPRI1*-capecitabine hastily attained stable equilibrium, and stable conformation of RMSD trajectory was observed throughout 300 ns of MD simulation (Fig. 2), and *LdmtPRI1*-ligand complexes display spatial binding patterns. It can be inferred from Fig. 3 that the ligand molecules maintain favourable molecular interactions in the *LdmtPRI1* binding pocket throughout the 300 ns simulation period.

Additionally, advanced MD simulations of MM-GBSA analysis (with 300 ns of run time) confirmed that the proposed *LdmtPRI1*-benfotiamine complex has a greater binding free energy (ΔGbind) score than *LdmtPRI*1-capecitabine (Table 3). The results of this study, which examined every detail from sequence levels to advanced structure dynamics, showed that benfotiamine may be able to block *LdmtPRI1* and certainly has antileishmanial properties. Benfotiamine, a synthetic S-acyl derivative of thiamine, inhibits the formation of advanced glycation end products, alleviating severe diabetic complications such as neuropathy, nephropathy and retinopathy^83^. Whereas, capecitabine, an antimetabolite, exhibits activity against numerous types of neoplasms (oesophagus, larynx, gastrointestinal and genitourinary tracts) that is metabolized to form compounds that interfere with the synthesis of DNA, RNA and proteins, resulting in inhibition of the proliferating cancerous cells and other hastily burgeoning cells, ensuing to their death^84^. *Leishmania* spp., like cancer cells, can persist in the host organism for an extended period, and certain enzymes targeted by anticancer treatments can also be utilized to form antileishmanial compounds^85,86^. For instance, miltefosine is the first and only oral drug for the treatment of VL and was initially developed for breast cancer treatment^87^.

In this investigation, benfotiamine and capecitabine were specifically selected for post-molecular docking analysis due to their elevated binding affinity against the *LdmtPRI1* binding site. The in-vitro study exclusively focused on benfotiamine and capecitabine, revealing a dose-dependent lethal effect against *Leishmania donovani* promastigotes, with IC_50_ values of 19.79 ± 0.67 μM and 12.40 ± 0.35 μM respectively as compared to Amphotericin B (12.02 ± 0.09 μM) (Fig. 7).

To validate the predicted computation results, the *LdmtPRI1* homologue was overexpressed in *E. coli*, and the corresponding polypeptide was purified via Ni^2+^ NTA chromatography and validated via SDS-PAGE (Supplementary Figure S6) to perform primase assay (Fig. 8A–E) and primase inhibition study using benfotiamine and capecitabine. Inhibition of primase activity was detected at drug concentrations of > 10 nM, signifying the inhibitory effects of the drugs used to hinder the activity of the primase enzyme (Fig. 9) with predicted IC_50_ values of 20.68 ± 0.033 nM and 15.27 ± 0.031 nM for benfotiamine and capecitabine respectively.

Discovering and developing new therapeutics to treat human parasitic infections is challenging. Identifying new chemical entities is the focus of de novo drug discovery. Possible benefits of drug repurposing techniques include making drug development more efficient and cutting costs. An intriguing review deliberating on drug repurposing has recently been published, providing an overview of multi-functional drugs that are effective in the treatment of leishmaniasis^88^ with specific emphasis on the development of drug repurposing strategies for multi-target strategies in order to identify potential candidates for the treatment of leishmaniasis. Significantly, the majority of leishmaniasis drugs currently on the market or in the early stages of drug discovery were initially intended for other therapeutic uses.

Consequently, based upon the analysis of our work as discussed above, we can surmise that benfotiamine and capecitabine might be the preferable and more secure medication over the currently accessible treatment option for *Leishmania* and could be a potential option to counter leishmaniasis. Hence, these drugs might be potent inhibitors that can be further experimentally tested to treat leishmaniasis.

## Conclusion

Repurposing drug molecules previously approved for treating specific diseases is a more effective, faster, and cost-effective disease treatment method. Overall, while it is certainly not the only answer, drug repurposing is an effective, relatively quick, and relatively inexpensive way to create critical new drugs, especially for neglected tropical infections like leishmaniasis. Our findings suggest that benfotiamine and capecitabine are probable drugs to counter leishmaniasis. Conversely, additional research using animal models would be required to conclude the safety and efficiency of these compounds. The present study's findings may provide crucial information for developing novel drugs to combat leishmaniasis.

### Supplementary Information

Supplementary Information.

## Data Availability

The datasets used and analyzed in this manuscript are available from the corresponding author on reasonable request.
