# Supplementary material for: Identification of potential inhibitor against Leishmania donovani mitochondrial DNA primase through in-silico and in vitro drug repurposing approaches
Source: Sci Rep. 2024 Feb 8;14:3246. doi: 10.1038/s41598-024-53316-5 (PMC10853515; doi:10.1038/s41598-024-53316-5)
Supplement: Supplementary file 1 — Supplementary Information. [file 41598_2024_53316_MOESM1_ESM.doc]

**Supplementary Data**

**“Identification of potential inhibitor against *Leishmania donovani* mitochondrial DNA primase through in-silico and in vitro drug repurposing approaches”**

Mitul Nath^1^, Deep Bhowmik^1^, Satabdi Saha^1^, Rajat Nandi^1^ and Diwakar Kumar^1^*

**Affiliation:**

^1^Department of Microbiology, Assam University, Silchar-788011, Assam, India

***Corresponding Author:**

Diwakar Kumar, PhD.,

Department of Microbiology, Assam University, Silchar-788011, Assam, India

E-mail: [diwakar11@gmail.com](mailto:diwakar11@gmail.com)

Tel: +91-8134080245

**Supplementary Figures**

**

**

**Figure S1.** (A) Predicted 3D structure of *LdmtPRI1* obtained from RoseTTAFold 9 (Robetta server) (B) Predicted active site of *LdmtPRI1* (Highlighted in red).

**
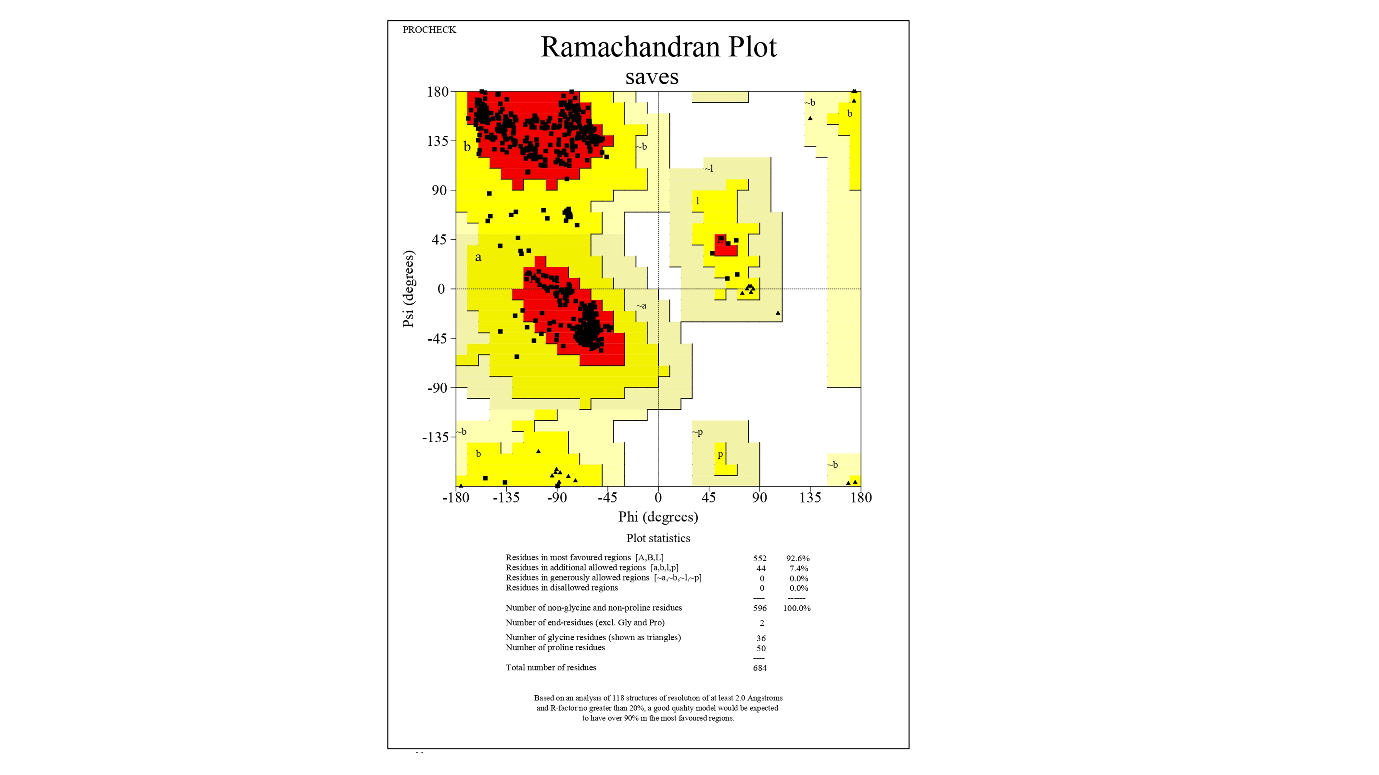
**

**Figure S2.** Ramachandran plot of *LdmtPRI1* generated by PROCHECK server.


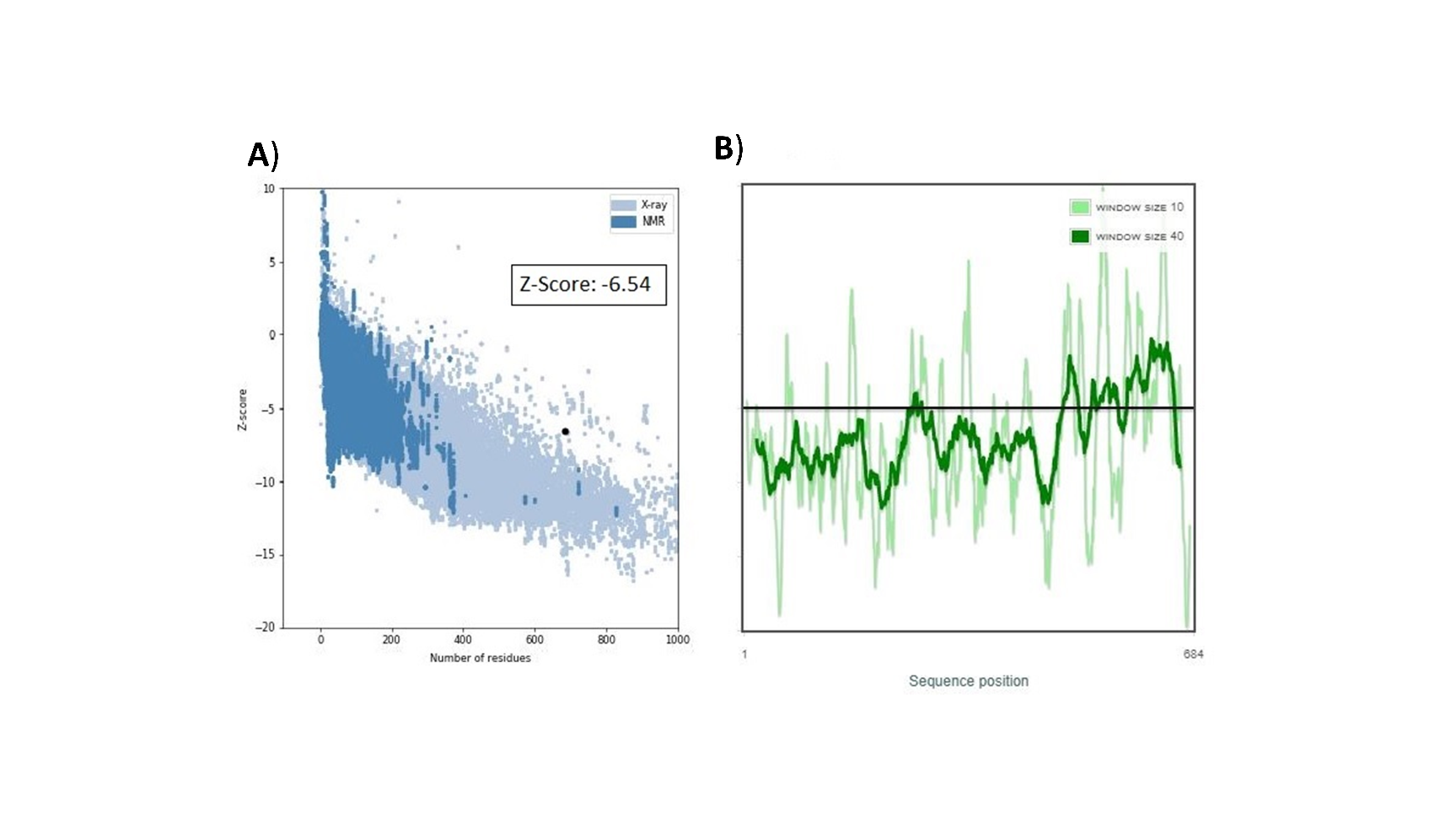


**Figure S3.** Z-Score and energy plot by ProSA. Left- Z-scores of all protein chains in PDB as determined by ProSa, which are confirmed by X-ray crystallography (light blue) or NMR spectroscopy (dark blue) with regard to their size. The z-score of *LdmtPRI1* is -6.54 and is highlighted with black dot. Right- Energy plot of *LdmtPRI1* as determined by ProSA. The thick line denotes average energy with a window size of 40 residues. The thin line denotes average energy over each 10 residues fragment in the background of the plot.

**

**

**Figure S4.** (A) 3D representation of molecular interaction of *LdmtPRI1* with ATP**,** here in figure yellow dot represents the H-bonds (B) 2D interaction between *LdmtPRI1* and ATP; H-bonds are represented in green colour.

**

**

**Figure S4.** (C) 3D representation of molecular interaction of *LdmtPRI1* with DCP**,** here in figure yellow dot represents the H-bonds (D) 2D interaction between *LdmtPRI1* and DCP; H-bonds are represented in green colour.


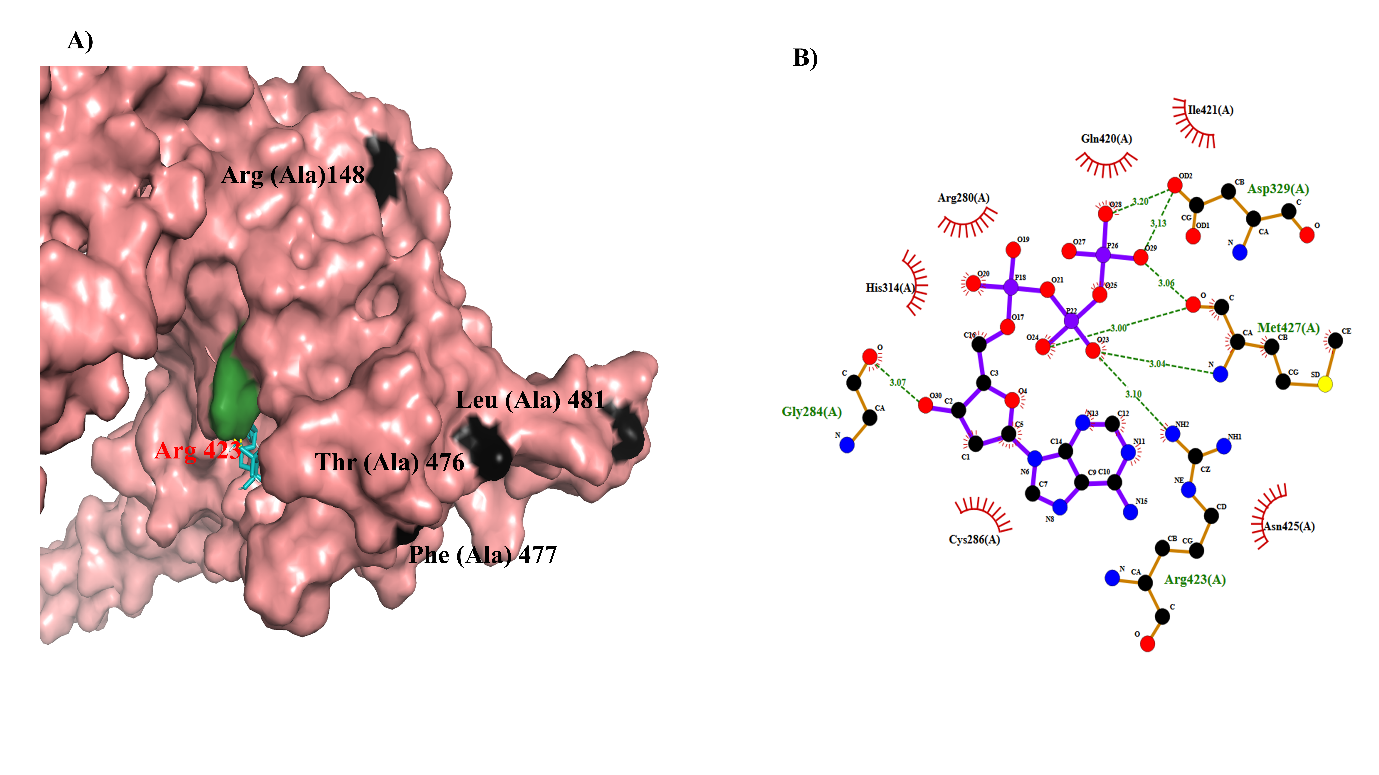


**Figure S5. (**A) 3D representation of dead variant of *LdmtPRI1* prepared by in-silico approach (Alanine mutation) highlighted in black. 3D interaction of mutant *LdmtPRI1* with ATP (highted in green) (B) 2D representation of interaction between mutant *LdmtPRI1* and ATP.


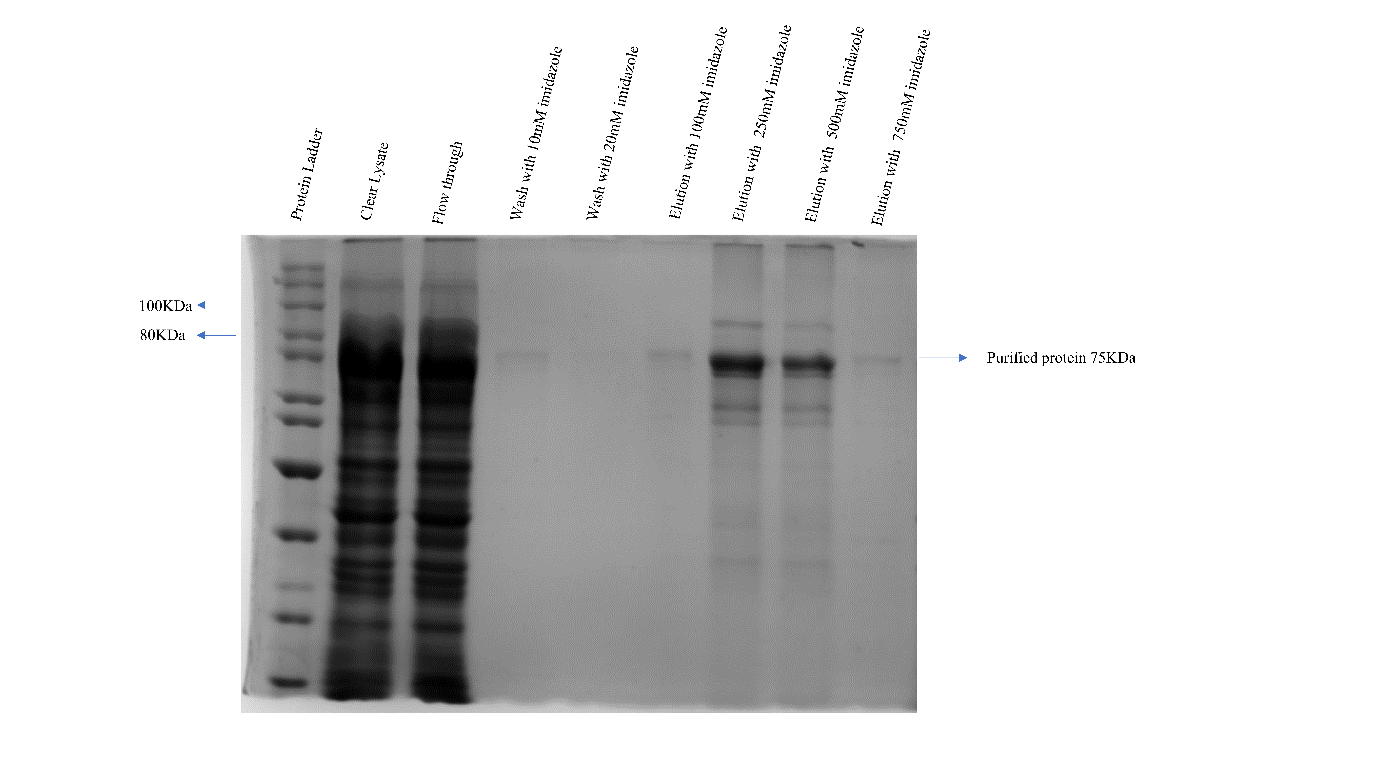


**Figure S6.** Coomassie stained SDS-PAGE analysis (12 % PAGE) of expression of pET-*LdmtPRI1* (His-*LdmtPRI1*). Expression and solubility analysis of His-*LdmtPRI1* protein at 16°C. Induction was carried out for 18hrs and purified by Ni^2+^-NTA column. 1- Long range protein ladder (Thermofischer), Lane 2- Clear lysate obtained post treatment of cell pellet with Ni^2+^-NTA resuspension buffer, Lane 3- Flow through passed through Ni^2+^-NTA column, Lane 4 and 5- Washing of column with 10 and 20 mM Imidazole respectively, Lane 6, 7,8 & 9 Elution of protein fraction using 100, 250,500 &750 mM Imidazole respectively.

**Supplementary Tables**

**Table S1.** Ramachandaran plot analysis of protein structure models in PROCHECK.

| Regions | Model-1 | Model-2 | Model-3 | Model-4 | Model-5 |
| --- | --- | --- | --- | --- | --- |
| Residues in most favoured regions [A, B, L] | 554 (93.0%) | 561 (94.1%) | 550 (92.3%) | 552 (92.6%) | 555 (93.1%) |
| Residues in additional allowed regions [a, b, l, p] | 42 (7.0%) | 34 (5.7%) | 45 (7.6%) | 44 (7.4%) | 40 (6.7%) |
| | Residues in generously allowed regions [âˆ¼a, âˆ¼b, âˆ¼l, âˆ¼p] | | --- | | 0 (0.0%) | 0 (0.0%) | 0 (0.0%) | 0 (0.0%) | 0 (0.0%) |
| Residues in disallowed region | 0 (0.0%) | 1 (0.2%) | 1 (0.2%) | 0 (0.0%) | 1 (0.2%) |
| Number of non-glycine and non-proline residues | 596 | 596 | 596 | 596 | 596 |
| Number of end-residues (excl, Gly and Pro) | 2 | 2 | 2 | 2 | 2 |
| Number of glycine residues | 36 | 36 | 36 | 36 | 36 |
| | Number of proline residues | | --- | | 50 | 50 | 50 | 50 | 50 |
| Total number of residues | 684 | 684 | 684 | 684 | 684 |

**Table S2. The ligand parameters to satisfy Lipinski’s rule of 5 and drug likeness score by Molsoft L.L.C.: Drug-likeness and molecular property prediction of the selected ligands after docking.**

| **Parameters** | **Compound name** | | | | | **Control drugs** | |
| --- | --- | --- | --- | --- | --- | --- | --- |
|  | Benfotiamine | Capecitabine | Febuxostat | Rolipram | Varespladib | AmpB | Miltefosine |
| **Mass** | 466.4 | 359.35 | 316.4 | 275.34 | 380.4 | 924.09 | 407.57 |
| **H-bond donor** | 4 | 3 | **1** | **1** | **3** | **0** | **1** |
| **H-bond acceptor** | 10 | 9 | 5 | 4 | 6 | 4 | 1 |
| **LOGP** | 2.63 | 1.01 | 3.72 | 2.62 | 2.38 | 0.71 | 5.67 |
| **Molar Refractivity** | 116.4 | 84.21 | 84.29 | 76.24 | 103.51 | 77.14 | 113.4 |
| **Drug likeness** | 1.32 | 0.4 | 0.05 | 0.87 | 0.62 | 0.16 | 0.83 |

**Table S3. ADMET property analysis of selected ligands along with the known drug (Amp B and Miltefosine) as control.**

| **Parameters** | **Compound name** | | | | | **Control drugs** | |
| --- | --- | --- | --- | --- | --- | --- | --- |
|  | Benfotiamine | Capecitabine | Febuxostat | Rolipram | Varespladib | AmpB | Miltefosine |
| Water Solubility | -2.79 | -3.135 | -3.46 | -3.06 | -3.51 | -2.94 | -6.15 |
| Caco2 Permeability | 0.34 | 0.255 | 1.03 | 1.29 | 0.77 | -0.59 | 1.05 |
| Intestinal Absorption | 60.66 | 68.08 | 93.46 | 92.82 | 54.82 | 0 | 92.02 |
| Skin Permeability | -2.74 | -2.75 | -2.733 | -3.00 | -2.74 | -2.74 | -2.72 |
| Pglycoprotein substrate | Yes | No | Yes | No | Yes | Yes | No |
| Pglycoprotein I Inhibitor | Yes | No | No | No | No | No | Yes |
| Pglycoprotein II Inhibitor | No | No | No | No | No | No | Yes |
| VDss (human) | -0.08 | -0.40 | -1.37 | 0.34 | -1.02 | -0.37 | 0.36 |
| Fraction unbound (human) | 0.24 | 0.39 | 0.23 | 0.23 | 0.15 | 0.54 | 0.16 |
| BBB Permeability | -1.84 | -1.45 | -0.61 | 0.22 | -0.50 | -2.06 | -0.17 |
| CNS Permeability | -3.75 | -3.32 | -2.2 | -2.83 | -2.85 | -3.72 | -3.19 |
| CYP2D6 Substrate | No | No | No | No | No | No | No |
| CYP3A4 Substrate | No | No | No | No | No | No | Yes |
| CYP1A2 Inhibitor | No | No | No | No | No | No | No |
| CYP2C19 Inhibitor | No | No | No | Yes | No | No | No |
| CYP2C9 Inhibitor | No | No | No | No | No | No | No |
| CYP2D6 Inhibitor | No | No | No | No | No | No | No |
| CYP3A4 Inhibitor | No | No | No | No | No | No | No |
| Total clearance | 0.06 | 1.05 | 0.31 | 1.01 | 0.38 | -1.50 | 1.11 |
| Renal OCT2 substrate | No | No | No | No | No | No | No |
| Ames Toxicity | No | No | No | No | No | No | No |
| Max. Tolerated Dose (Human) | 0.51 | 1.06 | 1.17 | 0.012 | 0.72 | 0.29 | 0.21 |
| hERG I Inhibitor | No | No | No | No | No | No | No |
| hERG II Inhibitor | No | No | No | No | No | No | Yes |
| Oral rat Acute Toxicity (LD50) | 2.31 | 2.46 | 2.32 | 2.44 | 2.76 | 2.51 | 2.66 |
| Oral rat Chronic Toxicity (LOAEL) | 2.19 | 2.40 | 1.802 | 1.25 | 2.48 | 2.04 | 0.23 |
| Hepatotoxicity | Yes | Yes | No | Yes | Yes | No | Yes |
| Skin Sensitization | No | No | No | No | No | No | Yes |
| Tetrahymena pyriformis toxicity | 0.28 | 0.29 | 0.31 | 1.38 | 0.29 | 0.28 | 0.31 |
| Minnow Toxicity | 1.48 | 2.89 | -0.43 | 1.59 | 1.39 | 11.26 | -1.84 |

**Table S4.** Spectrometry absorbance reading of primase optimization assay

| Divalent metal salts | Absorbance at 650nM | | | Standard deviation |
| --- | --- | --- | --- | --- |
|  | Absorbance 1 | Absorbance 2 | Absorbance 3 |  |
| MgCl_2_ | 3.38 | 3.43 | 3.19 | ±0.127 |
| MnCl_2_ | 2.68 | 2.51 | 2.56 | ±0.087 |
| CaCl_2_ | 1.42 | 1.26 | 1.37 | ±0.082 |
| ZnCl_2_ | 2.19 | 2.06 | 2.32 | ±0.260 |
| Time period in minutes |  | | | |
| 0 | 0.02 | 0.10 | 0.07 | ±0.040 |
| 10 | 0.18 | 0.11 | 0.22 | ±0.056 |
| 20 | 0.31 | 0.38 | 0.36 | ±0.036 |
| 30 | 0.48 | 0.53 | 0.57 | ±0.045 |
| 40 | 0.45 | 0.38 | 0.51 | ±0.065 |
| 50 | 0.39 | 0.31 | 0.28 | ±0.057 |
| 60 | 0.36 | 0.27 | 0.41 | ±0.071 |
| 70 | 0.29 | 0.22 | 0.19 | ±0.051 |
| M13mp18 ssDNA (uM) |  | | | |
| 0 | 0.089 | 0.077 | 0.069 | ±0.010 |
| 0.25 | 0.253 | 0.247 | 0.238 | ±0.007 |
| 0.5 | 0.479 | 0.465 | 0.486 | ±0.010 |
| 0.75 | 0.685 | 0.665 | 0.693 | ±0.014 |
| 1 | 0.782 | 0.802 | 0.792 | ±0.010 |
| 1.25 | 0.775 | 0.781 | 0.789 | ±0.007 |
| 1.5 | 0.752 | 0.745 | 0.734 | ±0.009 |
| Buffers and their pH |  | | | |
| Tris 6.8 | 2.13 | 2.07 | 2.19 | ±0.060 |
| Tris 7.5 | 2.51 | 2.57 | 2.67 | ±0.081 |
| Tris 8.0 | 2.49 | 2.43 | 2.31 | ±0.092 |
| HEPES 6.5 | 0.96 | 1.12 | 1.07 | ±0.082 |
| HEPES 7.5 | 2.58 | 2.63 | 2.66 | ±0.040 |
| MOPS 6.0 | 1.46 | 1.51 | 1.54 | ±0.041 |
| MOPS 6.5 | 1.92 | 1.83 | 1.98 | ±0.075 |
| CAPS 8.5 | 2.97 | 2.86 | 2.91 | ±0.055 |
| CAPS 8.8 | 3.74 | 3.81 | 3.93 | ±0.096 |
| CAPS 9.9 | 3.43 | 3.35 | 3.52 | ±0.085 |
| NTPs  (uM) |  | | | |
| 0 | 0.093 | 0.078 | 0.087 | ±0.007 |
| 50 | 0.247 | 0.243 | 0.249 | ±0.003 |
| 100 | 0.375 | 0.384 | 0.397 | ±0.011 |
| 150 | 0.369 | 0.358 | 0.351 | ±0.009 |
| 200 | 0.348 | 0.341 | 0.332 | ±0.008 |

**Table S5. Spectrometry absorbance reading of primase inhibition assay using drugs at different concentrations along with appropriate negative and positive controls**

| Drug used | Benfotiamine  (nM) | Absorbance at 650nm | | | Standard deviation |
| --- | --- | --- | --- | --- | --- |
|  |  | Absorbance 1 | Absorbance 2 | Absorbance 3 |  |
|  | 500 | 0.495 | 0.541 | 0.532 | ±0.024 |
|  | 250 | 0.734 | 0.802 | 0.818 | ±0.045 |
|  | 100 | 1.108 | 1.091 | 1.126 | ±0.018 |
|  | 50 | 1.281 | 1.192 | 1.247 | ±0.045 |
|  | 20 | 1.437 | 1.393 | 1.466 | ±0.037 |
|  | 10 | 1.593 | 1.526 | 1.581 | ±0.036 |
|  | Capecitabine  (nM) | | | | |
|  | 500 | 0.301 | 0.286 | 0.338 | ±0.027 |
|  | 250 | 0.704 | 0.728 | 0.752 | ±0.024 |
|  | 100 | 0.982 | 1.073 | 1.019 | ±0.046 |
|  | 50 | 1.068 | 1.105 | 1.052 | ±0.027 |
|  | 20 | 1.109 | 1.172 | 1.115 | ±0.035 |
|  | 10 | 1.234 | 1.197 | 1.268 | ±0.035 |
| 0.1% DMSO (Negative control) | | 2.397 | 2.336 | 2.407 | ±0.025 |
| PRIMASE (Positive control) | | 2.402 | 2.389 | 2.438 | ±0.026 |
